# An international tool to measure perceived stressors in intensive care units: the PS-ICU scale

**DOI:** 10.1186/s13613-021-00846-0

**Published:** 2021-04-10

**Authors:** Alexandra Laurent, Alicia Fournier, Florent Lheureux, Maria Cruz Martin Delgado, Maria G. Bocci, Alessia Prestifilippo, Pierre Aslanian, Julie Henriques, Sophie Paget-Bailly, Jean-Michel Constantin, Guillaume Besch, Jean-Pierre Quenot, Amelie Anota, Belaid Bouhemad, Gilles Capellier

**Affiliations:** 1 Psy-DREPI Laboratory EA 7458, University of Bourgogne Franche-Comté, Dijon, France; 2 Department of Anaesthesiology and Critical Care Medicine, University Hospital of Dijon Bourgogne, Dijon, France; 3 MSHE Ledoux, University of Bourgogne Franche-Comté, Dijon, France; 4 Laboratory of Psychology EA3188, University of Bourgogne Franche-Comté,, Dijon, France; 5 Hospital Universitario de Torrejón, Intensive Care Unit, Madrid, Spain; 6 School of Medicine, Universidad Francisco de Vitoria, Madrid, Spain; 7 Dipartimento di Scienze dell’Emergenza, Anestesiologiche e della Rianimazione, Rome, Italy; 8 Fondazione Policlinico Universitario Agostino Gemelli, IRCCS, Rome, Italy; 9 Intensive Care Unit of Centre Hospitalier de l’Université de Montréal, Montreal, QC Canada; 10 Methodology and Quality of Life in Oncology Unit (INSERM UMR 1098), University Hospital of Besançon, Besançon, France; 11 Department of Anaesthesiology and Critical Care, Pitié-Salpêtrière Hospital, Sorbonne University, GRC 29, AP-HP, DMU DREAM, Paris, France; 12 Department of Anesthesiology and Intensive Care Medicine, University Hospital of Besançon, University of Franche-Comte EA3920, Besançon, France; 13 Service de Médecine Intensive-Réanimation, University Hospital of Dijon Bourgogne, Dijon, France; 14 Medical Intensive Care Unit, University Hospital of Besançon, University of Franche-Comte EA 3920, Besançon, France

**Keywords:** Intensive care unit, Health care professionals, Job stress, PS-ICU scale

## Abstract

**Background:**

The intensive care unit is increasingly recognized as a stressful environment for healthcare professionals. This context has an impact on the health of these professionals but also on the quality of their personal and professional life. However, there is currently no validated scale to measure specific stressors perceived by healthcare professionals in intensive care. The aim of this study was to construct and validate in three languages a perceived stressors scale more specific to intensive care units (ICU).

**Results:**

We conducted a three-phase study between 2016 and 2019: (1) identification of stressors based on the verbatim of 165 nurses and physicians from 4 countries (Canada, France, Italy, and Spain). We identified 99 stressors, including those common to most healthcare professions (called *generic*), as well as stressors more specific to ICU professionals (called *specific*); (2) item elaboration and selection by a panel of interdisciplinary experts to build a provisional 99-item version of the scale. This version was pre-tested with 70 professionals in the 4 countries and enabled us to select 50 relevant items; (3) test of the validity of the scale in 497 ICU healthcare professionals. Factor analyses identified six dimensions: lack of fit with families and organizational functioning; patient- and family-related emotional load; complex/at risk situations and skill-related issues; workload and human resource management issues; difficulties related to team working; and suboptimal care situations. Correlations of the PS-ICU scale with a *generic* stressors measure (i.e., the Job Content Questionnaire) tested its convergent validity, while its correlations with the Maslach Burnout Inventory-HSS examined its concurrent validity. We also assessed the test–retest reliability of PS-ICU with intraclass correlation coefficients.

**Conclusions:**

The perceived stressors in intensive care units (PS-ICU) scale have good psychometric properties in all countries. It includes six broad dimensions covering *generic* or *specific* stressors to ICU, and thus, enables the identification of work situations that are likely to generate high levels of stress at the individual and unit levels. For future studies, this tool will enable the implementation of targeted corrective actions on which intervention research can be based. It also enables national and international comparisons of stressors’ impact.

**Supplementary Information:**

The online version contains supplementary material available at 10.1186/s13613-021-00846-0.

## Background

In the intensive care unit (ICU), healthcare professionals are faced with extreme situations, such as the constantly technological change, the end-of-life challenges, issues of organ retrieval or an epidemic crisis [1]. Assessed by individuals as situations that weaken or exceed their resources [2], occupational stressors can lead to burnout [3]. Accordingly, the prevalence of burnout in the ICU varies from 25 to 45% [4]. The considerable socio-economic costs resulting from burnout rank it among the most widely studied psychopathological impacts of work stress [4–9], along with anxiety, depression, moral distress [3, 9, 10] and suicide [11].

Assessing stressors with a view to preserving and supporting the mental health of professionals remains essential [3, 12]. However, the tools used to identify stressors remain inadequate, and the question of their relevance arises [13]. Indeed, as highlighted in a recent systematic review [14], available scales used in the field of ICU stress research lack metrological validity, or do not cover all the relevant ICU stressors. Out of 102 published studies (1997–2017) and dealing explicitly with the identification of ICU stressors, 35% of the studies used non-validated scales and 36% used *generic* scales, i.e., not specific to the ICU work environments (e.g., Job Content Questionnaire (JCQ) [15], Effort-Reward Imbalance [16]). Moreover, of the 22 ICU-*specific* scales, only two are validated: one about issues relevant to patient safety (Safety Attitudes Questionnaire-ICU [17]) and the other concerning the stress among nurses only (ICU nursing stress audit [18]).

Therefore, this paper presents the construction and validation in a large range of intensive care professionals of the perceived stressors in intensive care units (PS-ICU) scale, a measure exploring both *specific* and *generic* stressors, in different languages (allowing comparisons between countries). This work was carried out in three successive phases: Phase 1 (2016)—identification of stressors; Phase 2 (2017)—elaboration and selection of scale items; Phase 3 (2018–2019)—test of the validity of the scale.

## Phase 1: identification of stressors

### Method

The study obtained approval from the University Hospital’s Health Management team and the Committee for the Protection of Persons (CPP Est II, Ref: API/2014/56; Ref: 14/21). Participants were informed of the nature and potential risks of the study and signed a written consent form.

### Participants

**Fig. 1:**
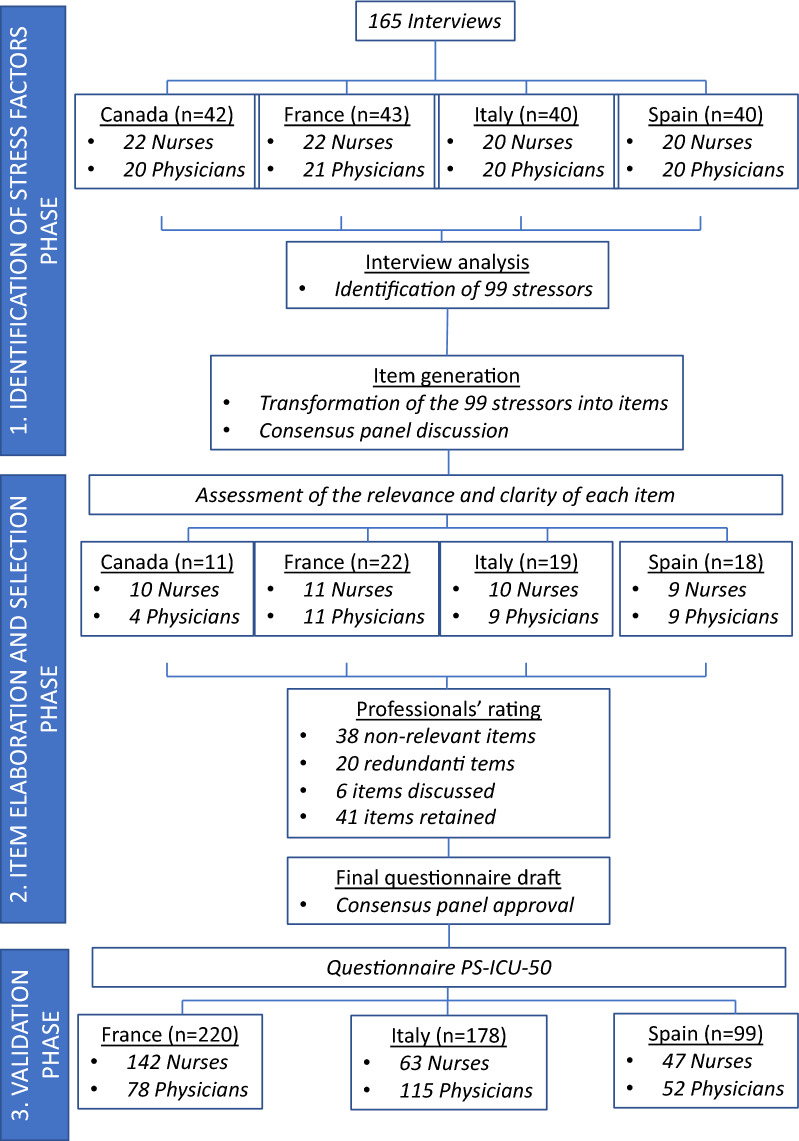
Study design—flowchart

Overall, for Phase 1, 165 volunteers, comprising physicians (junior and senior) and nurses (experienced and less experienced) from 6 University Hospitals in 4 countries (Canada (French speaking), Spain, Italy, France) were interviewed to identify stressors in the medical and surgical ICU settings (Fig. 1—flowchart). Based on the recommendations of Braun and Clarke [19] to ensure the basic representativeness of our sample in each country, we defined a minimal numbers of interviews (*n* = 20) per occupational categories (medical staff versus nursing staff) and per level of experience (senior/experienced professionals versus junior/early career professionals). Therefore, when crossing these two variables, we obtained 2 groups of participants: 20 physicians (10 seniors and 10 juniors), 20 nurses (10 experienced nurses (+ 1 year of experience) and 10 less experienced nurses (< 1 year of experience)). In addition, to account for the potential influence of gender, we included men and women in each group. See Table 1 for more information on the sample. To ensure correct sampling, recruitment of participants was done on site over a 1-month period by a research psychologist.

**Table 1:** Characteristics of the sample for Phase 1

	Canada	Spain	France	Italy	Total
Number of participants	42	40	43	40	165
Nurses	22 (52.4)	20 (50)	22 (51.2)	20 (50)	84 (50.9)
Physicians	20 (47.6)	20 (50)	21 (48.8)	20 (50)	81 (49.1)
Female/male ratio	20/22	22/18	26/17	26/14	94/71
Mean age	38.7 ± 10.9	38.1 ± 8.8	36 ± 7.5	33.1 ± 6.8	36.4 ± 8.8
Mean years since professional qualification	10.5 ± 10 (median: 8; IQR = 2.6–19)	13.1 ± 8.1 (median: 10.8; IQR: 8.2–18.8)	6 ± 5.7 (median: 3.8; IQR: 3–7)	8.5 ± 5.9 (median: 7.5; IQR: 3.9–11.8)	9.5 ± 8 (median: 7; IQR: 3–13.6)
Mean years in ICU	7.7 ± 4.6 (median: 5; IQR: 3.5–8)	8.6 ± 9.3 (median: 4; IQR: 1.3–14)	5.1 ± 5.8 (median: 2.5; IQR: 1.1–6)	8.2 ± 6.8 (median: 5.8; IQR: 2.8–14)	7.3 ± 7.8 (median: 4; IQR: 1.5–10.9)

Data represents *n* (%) or means ± SD; IQR, interquartile range (Q1–Q3)

### Procedure

We set up a French expert panel (five physicians, three psychology researchers, one psychologist, two nurses, two nursing managers, one epidemiologist, and two statisticians) whose mission was to coordinate data collection, centralize and pool the data and carry out a methodological framing of the research for all countries.

Professionals participated in an individual clinical interview conducted by a psychology researcher based in the country of the study. Interviews took place during their working time and in a dedicated small room within their unit. Only one open-ended question was asked: “What situations do you find difficult to bear in your work?” Based on the response received, the psychologist prompted the professionals to clarify problematic situations or to explore other difficult situations. The interviews lasted approximately 30 min and were audio-recorded and transcribed.

### Interview analysis

The French expert panel wrote methodological guidelines for researchers in other countries to carry out inductive thematic analysis based on the guidelines of Clarke and colleagues [20]. Meetings to harmonize practices were held throughout the data analysis process. In each country, after the full transcription of interviews in their original language, the texts were read thoroughly by the native psychologist researchers involved in the study to carry out the analysis. Using NVivo 10 software, the researchers generated codes in the form of stressors with a short description summarizing the idea conveyed. In each country, psychology researchers and physicians involved in the research discussed the preliminary codes identified, and reviewed, for each theme, the extracted verbatim. As a result, some codes were collapsed, and others were formed. The codes were refined with an accompanying narrative to summarize the central idea of each theme. After the coding process, a table was edited with each of the codes and one or two verbatim quotes to illustrate them. All the Italian, Spanish, French and French–Canadian qualitative results (e.g., codes, quotes) were put together by the French team and were translated into English by a native translator (and back translated by another bilingual translator to correct discrepancies). The French expert panel reviewed all codes identified in the four countries. They decided to merge some factors and rename others to obtain a single consensual typology of stressors. This expert panel then grouped all the stressors into broad categories guided by the literature [14], and complemented, in case of ambiguities, by the experience of clinicians.

## Results

Overall, 84 nurses (less experienced and experienced) and 81 physicians (junior and senior) were interviewed (94 women and 71 men; average age: 36.4 ± 8.8 years) (Table 1).

We identified 99 stressors (Additional file 1: Table S1). These were grouped into seven main categories of stressors: (1) significant workload pressure, (2) management of complex/at risk situations, (3) challenges related to one’s personal life, (4) dealing with ethical and moral-related situations, (5) problematic situations with patients and relatives, (6) conflicts with members of the healthcare team, and (7) lack of resources.

## Phase 2: item elaboration and selection

### Method

#### Participants

During a 4-month period, 70 voluntary professionals working in the same 6 ICUs as in Phase 1 completed the provisional 99-item PS-ICU scale (Fig. 1—flowchart). We paid attention to diversify and balance the profiles of professionals within each country.

### Procedure

The French expert panel transformed the 99 stressors identified during Phase 1 into 99 items by taking into account several criteria: content validity, relevance, clarity, fidelity to the qualitative content and redundancy. The expert panel also worked on the development of the instructions and response scale. Choices were made on the basis of consensus within the group and were also submitted for validation to collaborators in other countries involved. Instructions and items were translated from English into each country’s language: French, Italian, and Spanish. Each participant thus completed the questionnaire in his/her own native language.

The questionnaire submitted to the 70 professionals asked them to indicate whether they had experienced any of the following work situations in the ICU (0 if “Never”) and if so, to indicate the extent to which these situations disturbed them (Not at all (1)/A little (2)/Rather (3)/Extremely (4)). For this phase, professionals also assessed the relevance of each item (Yes/No) and, if yes, their respective importance (Not at all (1)/A little (2)/Fairly (3)/Very (4)). They ended the survey with a debriefing questionnaire containing open questions about the possible redundancy, disturbance and intrusiveness of items, as well as questions on the clarity of the instructions and presentation of the scale. The scale was completed online by ICU professionals.

### Analysis

Decision rules for deleting the items were formulated according to standard criteria [21]. Each item with at least one of the following criteria met was deleted:

- Floor effect (percentage of response in categories “0” and “1”) ≥ 50%
- Ceiling effect (percentage of response in category “4”) ≥ 50%
- Mean score < 1.5
- Missing data > 20%
- Relevance (Yes/No): < 70% of “Yes.”
- Importance (Not at all/A little/Fairly/Very): > 20% of “Not at all”
- Clarity (Yes/No): < 70% of “Yes”.

The expert panel also used open questions on redundancy and disturbance of the items.

## Results

The provisional version of the scale was tested between January and September 2017 on 70 ICU professionals (44 women and 26 men; average age: 35.65 ± 8.99 years), including 30 physicians (junior and senior) and 40 nurses (less experienced and experienced) (Table 2).

**Table 2:** Characteristics of the sample for Phase 2

	Total
Number of participants	70
Canada	11 (15.7)
Spain	18 (25.7)
France	22 (31.4)
Italy	19 (27.2)
Occupational status	
Nurses	40 (57.1)
Physicians	30 (42.9)
Female/male ratio	44/26
Mean age	35.65 ± 8.99
Mean years since professional qualification	9.8 ± 7.7 median: 8; IQR: 4.7–12.25)
Mean years in ICU	8.7 ± 7.6 median: 7.1; IQR: 3–12)

Data represents *n* (%) or means ± SD; IQR, interquartile range (Q1–Q3)

This pre-test allowed us to create a reduced and revised version of the scale. The instructions were slightly modified. The term “disturbed” was replaced by “stressed”. The Likert scale remained in a 5-point format. However, the headings of this scale were slightly modified from “*No*” to “*I have not experienced this situation*” (0) and from “*Yes. Faced with this situation, I was *(*not, little, rather, extremely*)* disturbed*” to “*I have experienced this situation and I was *(*not *(1)*, little *(2)*, rather *(3)*, extremely *(4))* stressed*”. A total of 38 items were kept with no discussion since they did not meet any deletion criteria and did not present any issues in the debriefing questionnaire. Conversely, 46 items met at least one deletion criteria and 30 items were discussed (the sum of items retained, discussed and meeting at least 1 of the deletion criteria exceeds 99 items because some items could meet at least 1 of the deletion criteria and also be discussed), mainly due to redundancy with other items, clarity of the questions, disturbance or too specific to medical activities. Results are displayed in the Additional file 1: Table S2. Finally, 49 items were removed, yielding a final 50-item stressors scale.

## Phase 3: validity of the PS-ICU scale

### Method

#### Participants

Professionals were recruited from 16 ICUs in France, Spain and Italy. At Time 1 (test), we recruited 497 voluntary professionals. Among them, 64.2% (*n* = 319) accepted to participate at Time 2 (retest) (Fig. 1—flowchart).

### Measures

For each national sample, the PS-ICU scale resulting from Phase 2 was used in the local native language (i.e., in French for France, Italian for Italy and Spanish for Spain).

To assess the convergent/divergent and concurrent validities of the PS-ICU scale, we used two other scales evaluating *generic* job stress (Job Content Questionnaire) and burnout (Maslach Burnout Inventory-Human Services Survey), respectively. Karasek’s Job Content Questionnaire (JCQ, [15]) is a widely used tool validated in each participating country [22–24]. This scale includes 29 items selected from the full version of JCQ. It evaluates four dimensions: (i) psychological job demands (9 items), (ii) decision latitude or job control (9 items), (iii) colleague support (6 items), and supervisor support (5 items). Each item was rated on a 4-point scale ranging from 1 (*strongly disagree*) to 4 (*strongly agree*).

The Maslach Burnout Inventory-Human Services Survey (MBI-HSS) [25], validated in each participating country [26–28], evaluates three dimensions typical of the burnout syndrome: emotional exhaustion (9 items), depersonalization (5 items) and [reduced] personal accomplishment (8 items). Each item was rated on a 7-point scale ranging from 0 (“*Never”*) to 6 (“*Every day”*).

For convergent validity, we expect to observe positive correlations between the total PS-ICU score and the “job demands” dimension score of the JCQ. For the divergent validity, we expect to observe negative correlations between the total PS-ICU score and scores on the “job control” and “social support” dimensions of the JCQ. For concurrent validity, we expect to observe positive associations between the PS-ICU score and scores on the “emotional exhaustion” and “depersonalization” dimensions, and negative associations with scores on the “personal accomplishment” dimension of the MBI-HSS.

### Procedure

Between December 2018 and October 2019, volunteer professionals received information via their professional mailbox inviting them to complete an online questionnaire via the Limesurvey platform. Participants completed the survey with two measurement times. At Time 1, participants provided socio-demographic data and completed the PS-ICU scale. In addition, to assess the concurrent and convergent validity of the scale, they also completed the MBI-HSS and the JCQ. Two weeks later, professionals were again individually invited to complete the questionnaire. They completed the PS-ICU scale again, to examine its test–retest reliability (time interval limited to 21 days). Since the experience of a particular stressful event between the two measurement times could have affected the stability of the stress experienced, participants were asked to answer questions about significant events they might have experienced between the two measurement times (e.g., divorce, significant event in my unit).

### Statistical analysis

Participants with more than five missing values for the entire PS-ICU scale were excluded. First, we performed descriptive analyses of the general characteristics of each sample (frequency, means and standard deviations).

To examine the structure of the 50-item PS-ICU scale, we compared results from factor analyses using three methods of extraction (principal component, principal axis and maximum likelihood) and two rotation techniques (Oblimin and Varimax), applied on different numbers of factors (suggested by Cattell’s scree test, Horn’s parallel analysis, the systematic review of Laurent et al. [14], and the qualitative results from the Phase 1). In the first round of analyses, four items (14,^1^ 22,^2^ 43,^3^ 47^4^) frequently had low loadings (< 0.30) across techniques (Additional file 1: Table S3). Therefore, we decided to remove these items during the subsequent second round. Since this round revealed low loadings (< 0.30) for three other items (30,^5^ 31,^6^ 39^7^) they were also removed to assess specific stressors. These analyses also revealed that the six-factor structure was most stable across extraction and rotation techniques. We finally selected the principal axis factoring method coupled with Oblimin rotation, because it yielded less cross-loading.

To complement the results of factor analysis, we also used Item Response Theory (IRT) models (Additional file 1: Table S4). We applied the Partial Credit Model for each identified factor to estimate an item difficulty parameter for each item on a logit scale [29]. In addition, we explored the adjustment of the model to the data with global and individual item-fit statistics and examined the fit residuals.

To calculate the score for each factor, we tolerated less than 20% missing values per factor. The score of each factor was obtained by averaging the scores of items that loaded on it in factor analysis. Since the 50 items obtained loadings above 0.40 on the first unrotated general stressor, we decided to use all of them to calculate the total PS-ICU score (mean score of all items). The higher the score is, the higher the perceived stress intensity.

Then, to observe whether there were differences between men and women on the one hand, and according to the occupational status on the other hand, on the different scores on the PS-ICU scale, we performed ANOVA with occupations or gender as between-subject independent variables and each PS-ICU score alternatively as dependent variables.

We next examined the internal consistency for each factor via Cronbach’s alpha and McDonald’s omega coefficients, and we examined the test–retest reliability using intraclass correlation coefficients for participants who experienced a significant event between the test and retest and those who did not (to estimate the sensitivity to change).

Finally, we used Pearson correlation coefficients and multiple regression analyses to examine the convergent/divergent validity of the PS-ICU scale with the four JCQ scores and the concurrent validity of the PS-ICU scale with the three scores of the MBI-HSS. In particular, multiple regression analyses estimated whether each stressor measured by PS-ICU was associated with the three burnout dimensions, while controlling for the effects of the other five stressors. If different patterns of results emerged for each PS-ICU stressor, this would establish their discriminant predictive validity, and therefore, the relevance of their differentiation.

The sample size was adequate for each statistical analysis [28, 30–34].

Statistical analyses were carried out with SPSS version 26.0, Jamovi 1.1.5 (Retrieved from https://www.jamovi.org), SAS software (version 9.4) (SAS Institute Inc., Cary, NC), RUMM2020 and PASS2020. The *p* value for statistical significance was set at *p* < 0.05. For more details of statistical analyses, see the Additional file 1: Box 1.

## Results

Overall, 497 participants were included in data analyses (331 women and 166 men; average age: 38.4 ± 9.94 years). Among them, 319 participated at Time 2 (retest) and 260 responded fully to the PS-ICU scale (167 women and 93 men; average age: 39.4 ± 10.3 years). The median time between both questionnaires was 18 days (interquartile interval 15–20) (Table 3).

**Table 3:** General characteristics of the participants in the Phase 3 validation study

	Overall population		France		Italy		Spain		*p* value
	(*n* = 497)		(*n* = 220)		(*n* = 178)		(*n* = 99)		
	*n*	%	*n*	%	*n*	%	*n*	%	
Gender									0.06
Male	166	33.40	78	35.45	65	36.52	23	23.23	
Female	331	66.60	142	64.55	113	63.48	76	76.77	
Age									< 0.001
Mean (SD)	38.4 (9.94)		34.3 (8.07)		42.3 (9.90)		40.6 (10.3)		
Missing	32		13		11		8		
Marital status									< 0.001
Single	113	23.35	38	17.51	61	35.41	14	14.74	
In a relationship	345	71.28	171	78.8	102	59.3	72	75.79	
Separated/divorced	23	4.75	8	3.69	8	4.65	7	7.37	
Widowed	3	0.62	0	0	1	0.58	2	2.11	
Missing	13		3		6		4		
Timing of work									< 0.001
Day	64	12.9	12	5.45	14	7.87	38	38.78	
Night	14	2.82	6	2.73	0	0	8	8.16	
Both	418	84.27	202	91.82	164	92.13	52	53.06	
Missing	1		0		0		1		
Job									< 0.001
Nurse	252	50.7	142	64.55	63	35.39	47	47.47	
Physician	245	49.3	78	35.45	115	64.61	52	52.53	
Work quota									0.002
Full time	405	81.98	170	77.27	160	91.43	75	75.76	
Part time	72	14.57	42	19.09	12	6.86	18	18.18	
Other	17	3.44	8	3.64	3	1.71	6	6.06	
Years since diploma									< 0.001
Mean (SD)	13 (9.57)		9 (7.63)		16 (9.54)		16 (10.29)		
Missing	7		6		1		0		
Years in ICU									< 0.001
Mean (SD)	10 (8.73)		7 (7.15)		12 (8.88)		14 (9.15)		
Missing	0		0		0		0		
Years since starting in this unit									< 0.001
Mean (SD)	8 (7.67)		5 (6.29)		9 (8.03)		11 (8.31)		
Missing	2		2		0		0		

ICU intensive care unit; SD standard deviation

The six-factor analysis (F1 to F6) (Table 4) accounted for 42.10% of the total variance. The KMO verified the sampling adequacy for the analysis (KMO = 0.933; individual KMO values ≥ 0.889 and ≤ 0.967). The results of IRT models showed no issues for most items (Additional file 1: Table S4). Item difficulty ranged from -0.96 to 0.58 logits, which indicates that no item was excessively easy or difficult to endorse. Only 1 item (#36) from F4 was likely to be redundant with other items from this factor with a fit residual < − 2.5. A differential item functioning was suspected for four other items (#6 and #15 from F1, #25 from F3 and #24 from F4) with a fit residual > 2.5. Two items obtained significant Bonferroni adjusted Chi-square values (#35 from F1 and #36 from F4), suggesting that they differed in their propensity to measure the stress associated with their target factor. Based on the literature and on the content of items that constitute each factor, the expert panel named F1 “Lack of fit with families and the organizational functioning” (10 items), F2 “Patient- and family-related emotional load” (10 items), F3 “Complex/at risk situations and skill-related issues” (5 items), F4 “Workload and human-resources management issues” (8 items), F5 “Difficulties related to team-working” (5 items), and F6 “Suboptimal care Situations” (5 items) (Table 4).

**Table 4:** Loading of the PS-ICU scale items from principal axis exploratory factor analysis with Oblimin direct rotation (items #14, #22, #30, #31, #39, #43 and #47 were preliminarily removed)

	Items	Factors (F)					
		F1	F2	F3	F4	F5	F6
9	Disagreement and/or lack of coordination with other units concerning a patient’s treatment	0.668					
10	Family conflict or disagreement concerning the patient’s treatment plan	0.554					
26	Family whose beliefs or lifestyle are contradictory with my values or the functioning of the unit	0.537					
27	Family’s misunderstanding of the gravity of the diagnosis or the prognosis of the patient	0.533					
42	Uncertainty concerning the diagnosis or the therapy project of the patient	0.486					
6	Shortage of beds in the unit	0.465					
4	Contradictory information given by other healthcare professionals to the family	0.464					
35	Family which does not trust me or does not trust the team	0.393					
41	Caring for a patient who should not be treated by the ICU	0.375					
15	Lack of support from the administration	0.302					
34	Death of a patient with whom I had developed special ties		0.626				
28	Series of patient deaths in the unit over a short period		0.615				
45	Having to announce a bad diagnosis to the patient or his/her family or be present when such a diagnosis is announced		0.581				
29	Patient who makes me think of someone close to me or of myself		0.540				
1	Socially isolated end-of-life patient or one with no immediate family		0.509				
49	Decision to stop or reduce treatment		0.476				
7	Families’ distress or emotions		0.402				
5	Caring for young patients or who have young children		0.363				
44	Patient suffering physically or psychologically		0.347				
18	Having to execute care tasks quickly in emergency cases			0.709			
40	Treating complex or serious pathologies			0.622			
16	Risk of error, fear of doing a poor job			0.596			
37	Having to perform tasks for which I have neither knowledge nor skills			0.509			
25	Patient who deteriorates in an unexpected or unexplained manner			0.405			
20	Working pace or working hours hardly compatible with family or social life				0.745		
23	Schedule changes, overtime				0.714		
36	Continuous and heavy workload				0.574		
50	Being on call or working nights				0.520		
48	Accumulated workloads resulting from clinical activity, training, research or teaching				0.405		
24	Working while experiencing difficult personal events		0.309		0.351		
32	Lack of staff				0.347		0.344
46	Lack of equality in the distribution of tasks among healthcare professionals				0.326		
13	Difficulty to find my place, have my skills recognized, or voice my opinion within the team					0.623	
38	Assessed or judged by the other members of the team					0.54	
17	Negative atmosphere prevailing in the team, gossip, rumors within the team					0.432	
3	Lack of recognition (from the patient, the family, the team, the hierarchy)					0.425	
21	Conflicts with members of the healthcare team	0.327				0.356	
8	Inadequate or under-equipped healthcare space or defective materials						0.44
33	Non-supportive, aggressive or delirious patient						0.394
12	Incomprehensible or unnecessary care relative to the patient’s situation						0.385
2	Colleague not doing his/her work properly						0.380
19	Plaintive patient who makes many requests			0.364			0.374
11	Too many professionals around the patient in an emergency situation						0.339
Eigen values			3.71	3	3.24	2.24	1.92
% of variance explained			8.62	6.98	7.55	5.2	4.47

For easy reading, all loading values < 0.30 were not reported. We present items in the order of the factors and according to their load on each factor. Some items had double saturation (#19, #21, #24, #32)

For all participants, all subscale scores and total scores had satisfactory internal consistency coefficients (*α* > 0.70). In addition, test–retest reliability for all subscale scores and total scores had satisfactory intraclass correlation coefficients (> 0.80) (Table 5).

**Table 5:** Results of test–retest reliability and validity of the PS-ICU scale analyses

	PS-ICU T1 scores (with Cronbach’s alpha in parentheses)						
	Factor 1 (*α* = 0.83; *ω* = 0.84)	Factor 2 (*α* = 0.83; *ω* = 0.83)	Factor 3 (*α* = 0.79; *ω* = 0.80)	Factor 4 (*α* = 0.81; *ω* = 0.82)	Factor 5 (*α* = 0.76; *ω* = 0.77)	Factor 6 (*α* = 0.80; *ω* = 0.80)	General factor (*α* = 0.95; *ω* = 0.95)
Correlation between factors (T1 test scores)							
Factor 2 (/4)	0.57^***^	–					
Factor 3 (/4)	0.33^***^	0.49^***^	–				
Factor 4 (/4)	0.61^***^	0.56^***^	0.44^***^	–			
Factor 5 (/4)	0.51^***^	0.49^***^	0.44^***^	0.54^***^	–		
Factor 6 (/4)	0.61^***^	0.57^***^	0.48^***^	0.58^***^	0.54^***^	–	
Test–retest reliability (intraclass correlations with T2 retest scores)							
Without significant event only (*n* = 197)	0.83	0.85	0.85	0.90	0.87	0.83	0.90
With significant event only (*n* = 67)	0.85	0.88	0.91	0.89	0.89	0.84	0.91
Convergent validity (correlations with the JCQ) (*n* = 233)							
Psychological demands (/36)	0.240^***^	0.241^***^	0.303^***^	0.438^***^	0.355^***^	0.333^***^	0.409^***^
Decisional latitude (/36)	− 0.149^**^	− 0.117^*^	− 0.118^*^	− 0.200^***^	− 0.258^***^	− 0.227^***^	− 0.228^***^
Colleagues’ support (/24)	− 0.191^***^	− 0.122^*^	0.050	− 0.182^***^	− 0.308^***^	− 0.120^*^	− 0.193^***^
Hierarchical support (/20)	− 0.084	− 0.082	− 0.082	− 0.214^***^	− 0.289^***^	− 0.141^**^	− 0.192^***^
Concurrent validity (correlations with the MBI-HSS) (*n* = 249)							
Emotional exhaustion (/54)	0.365^***^	0.386^***^	0.356^***^	.586^***^	0.469^***^	0.324^***^	0.539^***^
Depersonalization (/30)	0.201^***^	0.067	0.208^***^	0.193^***^	0.193^***^	0.172^***^	0.226^***^
Personal accomplishment (/48)	− 0.094	− 0.004	− 0.207^***^	− 0.085	− 0.195^***^	− 0.117^*^	− 0.143^**^

Factor 1 = lack of fit with families and the organizational functioning, Factor 2 = patient- and family-related emotional load; Factor 3 = complex/at risk situations and skill-related issues; Factor 4 = workload and human resource management issues; Factor 5 = difficulties related to team working; Factor 6 = suboptimal care situations

For the PS-ICU scale, the higher the factor scores are, the more intense the stress

For the JCQ dimensions, the higher the scores are, the higher the levels of demand, flexibility, and support

For the MBI-HSS dimensions, the higher the scores on emotional exhaustion and depersonalization dimensions, the more the scores reflect burnout. Conversely, the higher the scores on personal accomplishment dimension, the more the scores reflect a low or non-existent state of burnout

^***^p < 0.001; ^**^p < 0.01; ^*^p < 0.05

Analyses of differences according to gender and occupational status for each type of stressors showed that women were more stressed than men (except for F1 and F6 scores, where differences were non-significant). There were also differences between occupations, physicians having higher scores than nurses for F1 and F5, whereas nurses reported higher stress for F6 (Additional file 1: Table S5 for more details).

All PS-ICU scores were, in general, positively associated with job demands and negatively with job control and the two social support dimensions of the JCQ (satisfactory convergent validity). Likewise, all PS-ICU scores were, in general, positively associated with emotional exhaustion and depersonalization, whereas they were negatively associated with personal accomplishment (satisfactory concurrent validity) (Table 5).

The results of the three multivariate linear regression analyses are reported in Table 6. When entering all stressors as independent variables, predictive models for emotional exhaustion (*F*(6,463) = 50.8, *p* < 0.001, *R*^2^ adjusted = 0.39), depersonalization (*F*(6,446) = 7.10, *p* < 0.001, *R*^2^ adjusted = 0.07), and personal accomplishment (*F*(6,414) = 6.22, *p* < 0.001, *R*^*2*^* adjusted* = 0.07) were significant. Emotional exhaustion scores were positively associated with F3 (*b* = 1.44, 95% Confident Interval (CI) [0.23, 2.64]), the F4 (*b* = 6.78, 95% CI [5.38, 8.18]) and F5 (*b* = 2.84, 95% CI [1.74, 3.94]), while F6 was negatively associated with this dimension, *b* = − 1.97, 95% CI [− 3.50, − 0.44]. Depersonalization scores were positively associated with F1 (*b* = 1.23, 95% CI [0.17, 2.30]) and F3 (*b* = 1.22, 95% CI [0.45, 1.99]), and negatively associated with F2 (*b* = − 1.57, 95% CI [− 2.58, − 0.56]). Personal accomplishment scores were positively associated with F2 (*b* = 2.25, 95% CI [0.89, 3.60]) and negatively associated with F3 (*b* = − 2.07, 95% CI [− 3.14, − 1.00]) and the F5 (*b* = − 1.43, 95% CI [− 2.40, − 0.45]).

**Table 6:** Results (beta coefficients and significance) from multiple linear regression analyses with emotional exhaustion, depersonalization and personal accomplishment as predicted criteria

Predictors (PS-ICU subscales)	Dependent variables (MBI-HSS scores)		
	Emotional exhaustion	Depersonalization	Personal accomplishment
F1 Lack of fit with families and organizational functioning	− 0.12	1.23^*^	− 0.61
F2 Patient- and family-related emotional load	0.73	− 1.57^**^	2.25^***^
F3 Complex/at risk situations and skill-related issues	1.44^*^	1.22^**^	− 2.07^***^
F4 Workload and human resource management issues	6.78^***^	0.59	0.23
F5 Difficulties related to team working	2.84^***^	0.56	− 1.43^**^
F6 Suboptimal care situations	− 1.97^*^	0.17	− 0.13

^***^p < 0.001; **p < 0.01; *p < 0.05

## Discussion

This study aimed to develop and validate a scale of perceived stressors specific to the ICU. Based on 165 interviews in 4 countries, we were able to make a large inventory of situations experienced as stressful by ICU professionals. These situations were then discussed among the expert panel to form a preliminary version of the 99-item scale, which was pre-tested with 70 ICU professionals. The instructions, the Likert scale and the items were then discussed again among the expert panel, resulting in the final, 50-item PS-ICU scale. The PS-ICU scale was then validated in three languages (French, Italian and Spanish). Analyses show that the scale has good psychometric properties. The study of the association between PS-ICU scores and MBI-HSS dimensions shows that the PS-ICU scale has satisfactory concurrent validity when using burnout as measured by the MBI-HSS as the external criterion. The final version of the scale in French, Spanish and Italian is provided in the Additional file 1: Box 2.

The 50-item PS-ICU scale is divided into 6 major stressors: (1) lack of fit with families and organizational functioning (10 items); (2) patient- and family-related emotional load (9 items); (3) complex/at risk situations and skill-related issues (5 items); (4) workload and human resource management issues (8 items); (5) difficulties related to team working (5 items); and (6) suboptimal care situations (5 items). It allows us to assess *generic* stressors, i.e., common with other healthcare professions (e.g., high job demands, problematic relationships with other professionals, lack of resources), and *specific* stressors to the ICU (e.g., management of complex/at risk situations, dealing with ethical and moral issues, problematic situations with patients and relatives). Numerous studies have shown that complexity of care, ethics and the relationship with the patient and family are dimensions of stress that shape the identity of the unit and refer to strong emotional dimensions [7, 35, 36]. Therefore, it is essential to be able to take them into account when evaluating stress in the same way as more *generic* stressors, which is not possible with current *generic* scales such as the JCQ [14].

Finally, the PS-ICU scale is the first scale developed from the discourse of ICU professionals in four different countries and validated simultaneously in three different languages. This process of cross-cultural construction and validation reduces the likelihood that the results are dependent on the language, culture and health system of a particular country. The strength of the scale is therefore that it can be used in a wide range of professionals (less or more experienced nurses, and junior and senior physicians) from different cultural backgrounds, facilitating comparisons between countries. Indeed, in view of the results of Phase 2 (evaluation by professionals of the relevance and importance of the items), it did not seem necessary to develop two different scales for physicians and nurses. In addition, more studies will be necessary to propose country-based cut-off scores to account for the weight of cultural norms. Finally, studies on the validity of the French version of the PS-ICU scale in French-speaking Canada will be necessary, as well as for the English version that we have provided in the Additional file 1: Box 2.

Our study has limitations. Primarily, the 50-item PS-ICU scale is still relatively long, especially for clinical practice. Making the scale more compact would be a welcome object of future research. Six items (#6, #15, #24, #25, #35 and #36) appear to be in need of improvement or elimination, in view of the results of the IRT (inter-individual differences, redundancy or weighting of items within factors). Moreover, statistical analyses reveal a heterogeneity of participant profiles between countries, which reduces the relevance of subgroup comparisons. However, because the objective was not to make international comparisons while controlling for the effect of other variables, this is a moderate limitation that would be lifted by future studies specifically dedicated to this question. Since participation in the study was on a voluntary basis, it remains possible that respondents’ willingness to participate was associated with the issue at stake (i.e., evaluation of job stressors). As a result, participants might not be fully representative of the entire target population and this possible self-selection bias may have somewhat reduced the generalizability of our results [37]. This limitation should be overridden by testing our scale (and replicating the results) on other samples from different countries and clinical settings.

## Conclusion

The PS-ICU scale exhibits good psychometric properties. This scale, constructed to cover *specific* and *generic* perceived stressors in the ICU, will be useful for both clinical practice and research. It will enable the identification of the most challenging professional situations, and thus, enable the implementation of corrective actions. It will also facilitate the assessment of the effectiveness of stress-related interventions within ICU.

## Supplementary Information

**Additional file 1:** **Table S1.** Qualitative results of Phase 1. **Table S2.** Suppressed and discuted items during Phase 2. **Table S3.** Comparisons between different methods of extraction (principal component, principal axis and maximum likelihood) and rotation techniques (Oblimin and Varimax) with six-factor structure and without items 22, 14, 43, 47. **Table S4.** Difficulties and fit statistics for the PS-ICU items from Item Response Theory models. **Table S5.** Mean comparisons (ANOVAs) according to between-subjects (occupation and gender) and within-subjects (stress factors) variables. **Box 1.** Details of statistical analysis. **Box 2.** PS-ICU scale English, French, Spanish and Italian versions.

## Data Availability

Data are available from the corresponding author on reasonable request.

## Footnotes

1 Noisy environment.

2 Not being able to communicate with the patient.

3 Lack of respect towards the patient (towards his wishes, his integrity, his situation, etc.).

4 Unsuitable or under-equipped family reception area.

5 Powerlessness or incompetence in supporting families.

6 Changes in the modalities of care or the therapy project depending on the physician responsible for the patient.

7 Time spent on administrative tasks at the expense of care giving time.
