# Supplementary material for: An international tool to measure perceived stressors in intensive care units: the PS-ICU scale
Source: Ann Intensive Care. 2021 Apr 10;11:57. doi: 10.1186/s13613-021-00846-0 (PMC8036172; doi:10.1186/s13613-021-00846-0)
Supplement: Supplementary file 1 — Additional file 1: Table S1. Qualitative results of Phase 1. Table S2. Suppressed and discuted items during Phase 2. Table S3. Comparisons between different methods of extraction (principal component, principal axis and maximum likelihood) and rotation techniques (Oblimin and Varimax) with six-factor structure and without items 22, 14, 43, 47. Table S4. Difficulties and fit statistics for the PS-ICU items from Item Response Theory models. Table S5. Mean comparisons (ANOVAs) according to between-subjects (occupation and gender) and within-subjects (stress factors) variables. Box 1. Details of statistical analysis. Box 2. PS-ICU scale English, French, Spanish and Italian versions. [file 13613_2021_846_MOESM1_ESM.docx]

**Supplementary material**

**Table 1.**

Qualitative results of Phase 1

| Thematic Analysis |  | Example |
| --- | --- | --- |
| **High Job demands** |  |  |
| Training/Research/Coaching | Responsible for training and mentoring a colleague | *If they make a mistake, we make it too. We often think about that* |
|  | Being obliged to keep up-to-date on new practices | *The chief physician is really demanding that we be up to date in all our recommendations, that we be regularly trained, that we go beyond our achievements and that we must always maintain up-to-date knowledge, learn, read on the subject* |
|  | Having to conduct both clinical and research activities in the hospital | *Having to do everything at the same time, and at the same time not perform, not perform very well. It’s disturbing.* |
|  | Research takes precedence over patient management in the department | *When a study is in progress, we take the risk of making the patient undergo unpleasant acts even when we are not sure that he will tolerate them, simply* [because we want] *to include him in a study.* |
|  | Being scorned when I am not doing research | *We have a department that is promoting researchers. As clinicians we feel like we’re like being sidelined.* |
|  | Accumulation of the hospital workload and the workload outside the hospital related to training, research and teaching. | *I don’t want to do non-care-related activities [...] I am forced to.* |
|  | Staff changes or welcoming new healthcare professionals in the department | *There’s a turnover that is still very regular, which is sometimes difficult to manage because we sometimes work with people who are far more inexperienced.* |
| Pace of work | Night shifts or night duty | *At a certain point it was a lot, uh, well I, well, it was especially after being on call, being on call is a rather stressful moment* |
|  | Alternating day and night shifts. | *Having to change, uh, changing shifts, morning, noon, evening, night, whatever, that's stressful too.* |
| Workload | Constant and uninterrupted heavy workloads | *Yesterday I left here at almost 11 o’clock. I didn’t get a call throughout the night, still, it was a very busy day. The next day I had a twelve-hour day, the day after that I was on call again, I think being on call seeks above all to stress, I work the equivalent of two out of two weekends; that's a lot* |
|  | Decreased activity in the department | *There are certain days like yesterday, my day was totally calm. [...] When you have three, four days of calm like that you’re not okay.* |
|  | Increased responsibilities associated with a change in status | *Responsibilities that are really this change of status where as a resident we have responsibilities but we always have the chief physician supervising. When we change status, we’re the last to make decisions.* |
| **Management of complex or risky situations** |  |  |
| Emergency care | Having to execute care tasks quickly in emergency cases | *I have never had to deal with a cardiac arrest, resuscitation on my own. I have always had a chief physician with me. That, that stresses me regardless.* |
|  | Knowing how to prioritize care in emergency situations | *In an intensive care unit, you have to cope with several critical situations at the same time, which stresses me a lot* |
|  | Being patients’ last chance service | *We feel some tension when we think, we’re the last link in the chain. If we fail, no one else will succeed.* |
|  | Patient whose condition gets worse unexpectedly and inexplicably | *He was seriously ill, he was going to die, and we were trying to treat him and things were getting complicated, we would treat and something else would crop up. In short, he embodied the failure of medicine. I couldn’t stand it anymore, I couldn't treat him* |
| Complexity of care and risks | Management of serious and/or complex pathologies | *Actually he is already so seriously ill, so it’s not, it’s that we don’t try to rationalize in the acute, urgent approach where there is something serious that can be decompensated despite, despite the interventions, it doesn’t make the situation better, and worse, it goes wrong.* |
|  | Uncertainty concerning the diagnosis | *It’s true that we don’t know, we often don’t know what he has, we don’t know what we’ll have to do, that can be a source of stress.* |
|  | Having to perform tasks for which I have neither knowledge nor skills | *We are asked to rapidly acquire knowledge but without necessarily having the necessary experience. So stressful situations because you’re left, well, you're alone on call and you have to intubate a patient quickly and sometimes you don’t necessarily feel that you have the skills, that’s another stressful element.* |
|  | Risk of error, fear of doing a poor job | *I think to myself, ‘Damn it’, these are mistakes that shouldn’t be happening. Sometimes I get a little scared, once, once the repercussions for the patient have passed and there is no…, and it’s over for him, that’s what counts most* |
| **Challenges related to one’s personal life** |  |  |
|  | Pace of work or working hours barely compatible with family or social life | *You’re stuck until 8 p.m., I can’t pick up the kids, it all seems, it’s always very complicated.* |
|  | Working while experiencing difficult personal events | *I really wasn’t at ease with myself, that’s clear, and that clearly affected my, my anxiety, my ability to care for the patient* |
| **Dealing with ethical and moral-related situations** |  |  |
| Observe divergences between norms, values and ethical principles | Colleagues who do not respect the patient | *I’ve already had to admonish a colleague, to say, ‘no, we don’t talk about that in front of the patient’.* |
|  | Changes in the modalities of care or the therapy project depending on the physician responsible for the patient | *We say, well, anyway, we can’t do anything for him, we leave what we’ve put in place, but we do nothing more. The doctor changes and suddenly, we start over, we intubate, put him on the machine, we fiddle* |
|  | Too many professionals around the patient in an emergency situation | *Difficult situations... where everyone is stressed, where there’s a really major patient emergency and then, there are many many people. I am asked to do everything at the same time.* |
|  | Colleagues’ incompetence or negligence | *It’s complicated when at night, we have residents without their chiefs who are lost, who haven’t graduated yet, who take the liberty of neglecting the patient, that’s intolerable.* |
|  | Non-dedicated colleagues | *They’ll take ages before they react, yet we see that we need them. The time to act is now, but we’re looking for them because they're not here.* |
|  | Colleagues overly distressed by the situation | *The stress of an obsessive-compulsive colleague is stressful for someone like me.* |
|  | Contradictory information given by other healthcare professionals to the family | *It’s the hardest thing of all time. Working with a patient who’s going to die. Because [...]. We give the family false hope when there’s nothing left.* |
|  | Powerlessness or incompetence in supporting families | *I’m not always able to reassure them... I don’t have all the answers to their questions or I don’t have time, I can’t spend hours and hours with them. Uh, I see sometimes, that people are not satisfied with the discussions that I have with them.* |
|  | No consideration of the suffering experienced by patients’ families by other members of the healthcare team | *I think that sometimes it’s too brutal, it lacks empathy, it lacks the use of simple terms.* |
| Inappropriate care/lack of sense | Dehumanize my patient when providing care | *It’s really, this technical aspect, [...] where’s the patient in there? Where’s the patient?* |
|  | Lack of decision regarding the patient's therapeutic project | *Often it will be discussions, a debate about a patient or a file that can last half an hour, and in the end, we still don’t know what to do, not to do. Everyone gives his opinion and says what he thinks, but it’s not constructive.* |
|  | Lack of consideration of the patient's will | *So often have patients’ words not been respected, we have had patients who clearly expressed that they did not want to us to go that far, but we do it because there is a family member who is unable to move forward.* |
|  | Having to announce a bad diagnosis to the patient’s family in a department other than the ICU | *We are often asked to solve clinical and relational problems. I feel like a shield for the hospital. We are often the only ones who have a solution and say things as they really are. We feel like those who always bring bad news, I am angry because I have to play a role that is not mine.* |
|  | Mismatch between the care provided at the request of the family and the wishes expressed by the patient. | *All because the person of trust said: well, we’d rather have him intubated, we really felt like we weren’t taking into account the patient’s opinion.* |
|  | Being unable to communicate with the patient | *Because sometimes they don't talk when they’re in the ICU, but anyway he wanted to, did I provide what he wanted or not, because now he can’t talk* |
|  | Unequal treatment of patients | *Uh, it’s very easy to say, uh, why you’re dead set against this patient, uh, he’s a homeless guy, he’s going to go back on the streets and it’s very shocking to hear that from an ethical point of view, everyone has the right to the same care.* |
|  | Taking care of a patient whose behavior or lifestyle have deteriorated his or her health | *I took care of her the whole of last week, then, although she was better, uh... she couldn’t have a transplant so the liver she had, she couldn’t have a transplant ever, because she was on drugs, because of this, because of that. So why did we do all this? It’s just... we treat someone who’s going to continue using drugs, then we have nothing more to offer, then we keep doing everything... these are things that, sometimes it’s...* |
|  | Caring for a patient whose should not be in the ICU | *This problem should have been resolved earlier, it’s not up to us... This problem, it should have been addressed, it should have been resolved, it should have been addressed much earlier* |
|  | Incomprehensible or unnecessary care relative to the patient’s situation | *In a coma, with a tube in his mouth, what’s the meaning of that? In my opinion, quality of life goes hand in hand with keeping someone alive.* |
| Decision to stop or limit treatment | Stop treatment | *It’s painful, it’s painful, yeah, it’s sad [...] it’s things that are also a bit difficult, to manage, you understand on the one hand, you understand, but on the other it’s difficult.* |
|  | Being isolated from the decision to stop or limit treatment | *At some point, I am the one who makes the decision, and I’m all alone. You feel a little lonely sometimes when making these decisions.* |
|  | Complexity of the decision to stop or limit treatment | *No one can make a decision anymore. You have to wait, you have to go up, go higher* i*n the hierarchy you have to go see a specific doctor, another doctor will go see another one.* |
| **Problematic situations with patients and relatives** |  |  |
| Patients’ inappropriate expectations or behaviors | Lack of recognition from the patient | *Mm, because yeah, actually, I, I feel like yeah, actually, we take care of him, when he gets out of here, we don't actually have much information, whereas, uh, I, I’d like to know, well, uh, (incomprehensible), no, well he left in great shape.* |
|  | Non-conciliatory, aggressive and/or delusional patients | *But the people really who are, uh, unpleasant to me while – to me, personally – while I try to do everything to make things go well, that’s what hurts me the most in the patient relationship.* |
|  | Patients who complain and have many requests | *A patient who is very demanding, who is restless, or even every two minutes you have to be in the room, in the room, or take care of everything, of a patient, the patient, at the end of the evening, we’re “out” as we say, we’re tired.* |
| Inappropriate family expectations or behaviors | Families’ verbal/physical violence | *His response to this distress was aggressiveness, in, denial, aggressiveness, in his responses and all that, [...] I said to myself, I'm all alone I’m in the middle of nowhere, that’s if the guy attacks me, I’d die, I wouldn’t be able to get out of it. That was a difficult experience.* |
|  | Being in contact with a family whose beliefs or lifestyle are contradictory with my values or the functioning of the unit | *Then, he had to wait for the patriarch who came from China. The one who was to make the decision, so the eldest son. Um, actually, after waiting, doing all the administrative tasks, this gentleman came. Then when he came, so in front of an assembly of about 30 people, the whole extended family which was there. And I was with the nurse.”* |
|  | Family which does not trust me or does not trust the team | *We always come back to the same problem of families who are really stressed, they only look at the patient board, they no longer even look at their loved one.* |
|  | Fear of legal proceedings | *They want to sue everyone, basically I know I didn’t make any medical mistakes, so in theory there’s no basis, but it’s always extra stress because we know that we may have to go to court and settle these things, [...] the fear of being prosecuted* |
|  | Family’s poor understanding of the severity of the patient's diagnosis or prognosis | *Another frustrating issue is when I feel that a family member does not want or cannot understand the patient’s real situation.* |
|  | Lack of family’s recognition for the team | *We don’t do it to get the patient’s or his family’s recognition because we never get it.* |
|  | Presence of families during care | *There are people who do not respect the, the, the, protocol. Well who, who, who impose themselves, well who, who do not respect the fact that during care procedures, they have to go back to the family waiting room. So that’s a little difficult to deal with because sometimes you have to enforce discipline.* |
|  | Having to deal with family conflict or disagreement concerning the patient’s treatment plan | *When families quarrel among themselves and I become a family psychologist or they disagree or find it difficult to come to an agreement, these are situations that I find difficult* |
|  | Spending more time with families than with patients | *Families who are very present, uh, who always want more information, who always want to go into the details that they don’t necessarily understand. That’s very stressful, because in the end we no longer have anything to say and they always want to know more.* |
| Having to deal with the patient's death/end of life | Socially isolated end-of-life patient or one with no immediate family | *It’s hard when there’s a patient, who dies, for instance, in ICU, attached to a tube and there’s no one, around, that’s, I find that horrible, I find it really distressing, it’s, yeah that’s, really distressing.* |
|  | Death of a patient with whom I had developed special ties | *We think about it more* [about patients] *when they were conscious when they arrived, we were able to talk with them, they told us a little about their way of life, their family, etc. And then, well of the sudden, we intubate them and unfortunately they die and yeah, we think about it more, we think more about them than when it’s a patient who arrives intubated and with whom we haven't had a relationship.* |
|  | Series of patient deaths in the unit over a short period | *Sometimes we have many patients who die in a row, we get a little sick and tired. So we’re less motivated. [...]. It feels like it’s somewhat useless, so that lowers commitment* |
|  | Caring for a patient after death | *You feel useless... […] it’s palliative care. [...] here palliative care, they don't want to hear about it, even if that’s what you do.* |
|  | Having to announce a diagnosis or a bad diagnosis to the patient | *Uh, announcing a bad diagnosis to the patient... that marks one.* |
| Having to face the distress or pain of the patient or family | Patient who makes me think of someone close to me or of myself | *It’s somewhat similar to what my father experienced and I was just doing the file, my eyes filled with tears and then I couldn’t shake it off, I know it’s a factor that affects me a lot.* |
|  | Having to announce a diagnosis or a bad diagnosis to the family | *When a patient has a bad diagnosis and I have to face my family.* |
|  | Having to announce a decision to limit or discontinue the use of active therapeutic substances. | *Decisions must be made about the discontinuation of therapy or even a discontinuation of care that must lead to death. With families who we’re unable to have easy, simple, pleasant communication, this is a situation that disturbs me very much, which I always find very difficult.* |
|  | Taking care of a patient I know personally | *I had to revive my colleague who I’ve known for fifteen years and who died in front of me, then, but finally he survived, that went well but just seeing him, and having had the utmost stress of resuscitating someone you know and that I’m, I had flashbacks that would wake me up at night for weeks.* |
|  | Caring for young patients and/or those who have young children | *A 30-year-old man who, no medical past, [...], thirty years, an 11-month-old daughter, his wife who is there, then, severe sepsis, uh, he died in my arms after erratic changes, the type of patient, I talk to him when he arrives, he speaks then six hours later he’s dead, [...] you don’t sleep, you’re at his bedside, you meet the family but when he passed away, I had to sit down for fifteen minutes.* |
|  | Patients suffering physically and/or psychologically | *You have to be honest he’s suffering, well, and, uh, very unstable, he was extubated, so he didn’t necessarily have, he wasn’t on morphine, he wasn’t taking anything, so, uh, that’s it.* |
|  | Performing care activities that are painful for the patient | *We would change the bandages and we would hear her screaming throughout. It was I who provided “sedation,” or analgesia, I would give her terribly high doses and despite everything she would scream because everything hurt her.* |
|  | Seeing the patient’s “damaged” body | *They were in a state, well it was (sighing), it was a vision of horror [...] road accidents, I don’t know why, it's systematic [...] they make my stomach turn a little.* |
|  | Having to deal with families’ distress or emotions | *I always fear that it will shatter and all, uh (silence +) and you know, emotions, emotions are just very, very, very strong, and it just becomes a cumbersome and difficult time.* |
| **Problematic relationships with other professionals** |  |  |
| Pressure from colleagues | Being assessed or judged by the other members of the team | *It is how others perceive our actions. It generates stress somewhat; well it generates some stress, [and] it can generate somewhat embarrassing situations because there is direct judgment in situations* |
|  | Competing with colleagues | *There’s some competition, the one who produces the most brings in the most.* |
| Conflict/lack of recognition | Difficulties in seeking help from a colleague, seeking a physician | *It’s very distressing to ask someone, to ask them for the solution.* |
|  | Conflicts with one or several members of the healthcare team | *I’ve had conflicts with nurses, and it was terrible, all our natural authority is undermined, our medical authority is undermined.* |
|  | Difficulty in finding one’s place, having one’s skills recognized, or voicing one’s opinion within the team | *At first, it’s difficult, you have to make your mark, you have to prove yourself.* |
|  | Lack of recognition for a job well done | *And especially no recognition when you do things right. I mean when you work, well all year long and things go rather well no one tells you anything, but the day you do something then they crush you.* |
|  | Devaluation of skills because of one’s gender or family status | *It’s a very misogynistic society, I was the only woman in the department, and I was told that I was going to do things in a particular way, that I was going to participate in this undertaking and that if not...* |
|  | Disagreement and/or lack of coordination with other units concerning a patient’s treatment | *I get annoyed when I have to explain that. [...]. Go and explain to a family that we’re not going to operate because the neurosurgeon doesn’t think it's necessary. I’d like the specialist who sets the limit to be the one to see the family.* |
|  | Negative atmosphere prevailing in the team, gossip, rumors within the team | *Internal conflicts can be cumbersome, it can be stressful, when you get somewhat caught up in things like that* |
| **Lack of resources** |  |  |
| Organizational | Inadequate space for downstream services | *Well, it’s really annoying because we were full, we had 15 occupied beds, three outgoing patients and no beds available in ancillary services. So we couldn’t get them out even though there was a surge* |
|  | Unequal distribution of patients | *Patients are forced on us, [...] the fact, for example, of seeing someone who has a considerably lighter workload than we who are running all over, and that she doesn’t offer us her help, well that’s frustrating.* |
|  | Shortage of beds in the unit | *For example, this sick person who dies on a stretcher with the family... And well, it makes me angry to have to answer instead of someone in administration.* |
|  | Unplanned and/or rapid discharge of a patient in order to accommodate a new patient | *Other stressful situations are, uh, when you have to get patients out quickly to make room for others who are waiting. It’s stressful because we don't get them out in the right way, under conditions that are somewhat limited, not too humane.* |
|  | Isolation at night | *Oh yes, yes, I’m always worried, I’m always worried about coming on a Sunday and being alone, having to take care of 20 patients.* |
|  | Insufficient time for patient management | *If I had been with a partner longer, I think I would have learned a lot more, because once you’re there, abandoned, you can’t ask questions* |
|  | Schedule changes, overtime | *Last-minute schedule changes, because it leads to changes in my personal life, which are also difficult to manage because I have kids to manage and, uh, last-minute schedule changes, I have to find someone to take care of them, so, yeah, it’s hard* |
|  | Staff shortages | *Many staff members were missing, especially at night, meaning that we were on compulsory overtime. We were under a great deal of stress because we didn’t know if we were going to, uh, leave at midnight. That, that year was a really difficult one.* |
|  | Time spent on administrative tasks at the expense of care activities | *We have to fill out a lot of paperwork at the expense of the patient [...] I no longer feel like... We... well, we don't pay attention to what's really important for professionals and then patients.* |
| Environmental | Noisy environment | *The other factor, I believe incredibly stressful in ICUs, is alarms.  There’s constant ringing, ringing, ringing, all the time, all the time* |
|  | Faulty or unavailable equipment | *At the material level. Because it’s true when the material isn’t working, or if it’s missing* |
|  | Inadequate or under-equipped healthcare space | *It’s not practical. The doors are a bit narrow, the corridors are very narrow and there are 90-degree turns, so when you have to go to the scanner, and the floors as well, the floor coating because to go to the scanner while pushing the stretcher, it doesn’t roll. It's a disaster!”* |
|  | Unsuitable or under-equipped space to receive families | *To receive families in rooms,* [hold] *interviews where there are no windows, to have broken beds...* |
|  | Lack of user-friendliness space | *There’s really a need for more time and space between healthcare professionals. [] to strengthen the team’s ties a little more, to get to know people a little better, to improve everyday life.* |
|  | Lack of a platform to express one's feeling at work | *I think we don’t have time, we never have time for discussions or debriefing... we never talk about our feelings and all that.* |
|  | Ambient temperature | *Um, I have to use sterile techniques, I have to put on two sets of clothes but it’s 40* [degrees] *in the room because the patient is losing heat so you have to keep him warm. So that’s difficult.* |
| Institutional | Having to constantly change units | *It’s the unknown to be in a hospital where you've never worked, never worked with people you don't know, a way of functioning that you don’t know.* |
|  | Economic and/or material constraints preventing optimal patient care | *Management doesn’t want to manage it* [training to learn how to set up artificial circulation]*. We don’t really know how it works. These are devices for which all staff should be trained. It’s the training system where you have to find the time, uh, you have to find the money.* |
|  | Uncertainty in the future of one’s career | *We are all on precarious fixed-term contracts which therefore are renewable from year to year but it is true that we cannot anticipate the future beyond a year since we don’t know what will become of us a year later.* |
|  | Lack of support from the administration | *A wide gap between the administration and the people on the ground, and often in some decisions. For example, the dress code, certain operating rules that seem very far from the reality on the ground, from healthcare professionals* [such as] *the fact that the staff is forced to do extra duty* [or] *that the cafeteria is closed on weekends. The element missing here is the hospital’s administration, that we go see them, we tell them well listen we have a problem, well they* [the administration] *are very timid.* |

**Table 2**

Supressed and discuted items during Phase 2

|  | **Item label** | **Criteria** | **Open questions** | **Final item** |
| --- | --- | --- | --- | --- |
| **1** | Socially isolated end-of-life patient or one with no immediate family |  |  | Socially isolated end-of-life patient or one with no immediate family |
| **3** | Contradictory information given by other healthcare professionals to the family |  |  | Contradictory information given by other healthcare professionals to the family |
| **5** | Caring for young patients or who have young children |  |  | Caring for young patients or who have young children |
| **8** | Families’ distress or emotions |  |  | Families’ distress or emotions |
| **10** | Unsuitable or under-equipped space to receive families |  |  | Unsuitable or under-equipped space to receive families |
| **13** | Family which does not trust me or does not trust the team |  |  | Family which does not trust me or does not trust the team |
| **16** | Disagreement and/or lack of coordination with other units concerning a patient’s treatment |  |  | Disagreement and/or lack of coordination with other units concerning a patient’s treatment |
| **17** | Family conflict or disagreement concerning the patient’s treatment plan |  |  | Family conflict or disagreement concerning the patient’s treatment plan |
| **18** | Too many professionals around the patient in an emergency situation |  |  | Too many professionals around the patient in an emergency situation |
| **19** | Incomprehensible or unnecessary care relative to the patient’s situation |  |  | Incomprehensible or unnecessary care relative to the patient’s situation |
| **20** | Difficulty to find my place, have my skills recognized, or voice my opinion within the team |  |  | Difficulty to find my place, have my skills recognized, or voice my opinion within the team |
| **21** | Powerlessness or incompetence in supporting families |  |  | Powerlessness or incompetence in supporting families |
| **24** | Noisy environment |  |  | Noisy environment |
| **28** | Lack of support from the administration |  |  | Lack of support from the administration |
| **29** | Risk of error, fear of doing a poor job |  |  | Risk of error, fear of doing a poor job |
| **30** | Having to execute care tasks quickly in emergency cases |  |  | Having to execute care tasks quickly in emergency cases |
| **33** | Plaintive patient who makes many requests |  |  | Plaintive patient who makes many requests |
| **35** | Working pace or working hours hardly compatible with family or social life |  |  | Working pace or working hours hardly compatible with family or social life |
| **37** | Conflicts with members of the healthcare team |  |  | Conflicts with members of the healthcare team |
| **40** | Not being able to communicate with the patient |  |  | Not being able to communicate with the patient |
| **41** | Schedule changes, overtime |  |  | Schedule changes, overtime |
| **47** | Working while experiencing difficult personal events |  |  | Working while experiencing difficult personal events |
| **48** | Patient who deteriorates in an unexpected or unexplained manner |  |  | Patient who deteriorates in an unexpected or unexplained manner |
| **52** | Family whose beliefs or lifestyle are contradictory with my values or the functioning of the unit |  |  | Family whose beliefs or lifestyle are contradictory with my values or the functioning of the unit |
| **54** | Family’s misunderstanding of the gravity of the diagnosis or the prognosis of the patient |  |  | Family’s misunderstanding of the gravity of the diagnosis or the prognosis of the patient |
| **55** | Series of patient deaths in the unit over a short period |  |  | Series of patient deaths in the unit over a short period |
| **58** | Patient who makes me think of someone close to me or of myself |  |  | Patient who makes me think of someone close to me or of myself |
| **60** | Changes in the modalities of care or the therapy project depending on the doctor responsible for the patient |  |  | Changes in the modalities of care or the therapy project depending on the doctor responsible for the patient |
| **61** | Lack of staff |  |  | Lack of staff |
| **67** | Non-supportive, aggressive or delirious patient |  |  | Non-supportive, aggressive or delirious patient |
| **69** | Death of a patient with whom I had developed special ties |  |  | Death of a patient with whom I had developed special ties |
| **73** | Negative atmosphere prevailing in the team, gossip, rumours within the team |  |  | Negative atmosphere prevailing in the team, gossip, rumours within the team |
| **81** | Continuous and heavy workload |  |  | Continuous and heavy workload |
| **82** | Having to perform tasks for which I have neither knowledge nor skills |  |  | Having to perform tasks for which I have neither knowledge nor skills |
| **85** | Shortage of beds in the unit |  | To discuss | Shortage of beds in the unit |
| **86** | Assessed or judged by the other members of the team |  |  | Assessed or judged by the other members of the team |
| **90** | Time spent on administrative tasks at the expense of care giving time |  |  | Time spent on administrative tasks at the expense of care giving time |
| **97** | Treating complex or serious pathologies |  |  | Treating complex or serious pathologies |
| **98** | Caring for a patient who should not be treated by the ICU |  |  | Caring for a patient who should not be treated by the ICU |
| **2** | Non-dedicated colleagues | Redundancy |  | Colleague not doing his/her work properly |
| **9** | Colleagues overly distressed by the situation | Redundancy |  |  |
| **15** | Colleagues’ incompetence or negligence |  | To group or suppress |  |
| **7** | Isolation at night | Redundancy |  | Being on call or working nights |
| **68** | Night shifts or night duty | Redundancy |  |  |
| **92** | Alternating day and night shifts. | Redundancy |  |  |
| **75** | Inadequate or under-equipped healthcare space |  |  | Inadequate or under-equipped healthcare space or defective materials |
| **12** | Faulty or unavailable equipment |  |  |  |
| **26** | Uncertainty concerning the diagnosis | Redundancy |  | Uncertainty concerning the diagnosis or the therapy project of the patient |
| **59** | Lack of decision regarding the patient's therapeutic project | Redundancy |  |  |
| **27** | Lack of recognition for a job well done |  |  | Lack of recognition (from the patient, the family, the team, the hierarchy) |
| **65** | Lack of family’s recognition for the team | Floor effect (≥ 50%) ; redundancy |  |  |
| **78** | Lack of recognition from the patient | Floor effect (≥ 50%) ; redundancy |  |  |
| **31** | Patients suffering physically and/or psychologically | Redundancy |  | Patient suffering physically or psychologically |
| **36** | Seeing the patient’s “damaged” body | Redundancy |  |  |
| **39** | Performing care activities that are painful for the patient | Redundancy |  |  |
| **4** | Unplanned and/or rapid discharge of a patient in order to accommodate a new patient | Redundancy |  | Lack of respect for the patient (against his/her wishes, his/her integrity, his/her situation, etc.) |
| **34** | Colleagues who do not respect the patient | Redundancy |  |  |
| **51** | Contradictory information given by other healthcare professionals to the family | Redundancy |  |  |
| **62** | Dehumanize my patient when providing care |  | Clarity |  |
| **87** | Lack of consideration of the patient's will | Redundancy |  |  |
| **89** | Unequal distribution of patients |  | To discuss | Lack of equality in the distribution of tasks among healthcare professionals |
| **79** | Accumulation of the hospital workload and the workload outside the hospital related to training, research and teaching. |  | Doctors specific | Accumulated workloads resulting from clinical activity, training, research or teaching |
| **91** | Having to conduct both clinical and research activities in the hospital |  | Doctors specific |  |
| **25** | Having to announce a decision to limit or discontinue the use of active therapeutic substances. | Floor effect (≥ 50%); redundancy | Doctors specific | Having to announce a bad diagnosis to the patient or his/her family or be present when such a diagnosis is announced |
| **57** | Having to announce a diagnosis or a bad diagnosis to the family |  | Doctors specific |  |
| **70** | Having to announce a bad diagnosis to the patient’s family in a department other than the ICU |  | Doctors specific |  |
| **80** | Having to announce a diagnosis or a bad diagnosis to the patient |  | Doctors specific |  |
| **14** | Being isolated from the decision to stop or limit treatment |  | Doctors specific | Decision to stop or reduce treatment |
| **6** | Spending more time with families than with patients | Floor effect (≥ 50%); relevance <70% | Clarity | Suppression |
| **11** | Lack of fairness regarding the care provided to patients | Relevance <70%; redundancy | Clarity | Suppression |
| **22** | Families’ verbal/physical violence | Floor effect (≥ 50%) |  | Suppression |
| **23** | Caring for a patient after death | Floor effect (≥ 50%) |  | Suppression |
| **32** | Being patients’ last chance service | Floor effect (≥ 50%); redundancy | Disturbance | Suppression |
| **38** | Fear of legal proceedings | Floor effect (≥ 50%) |  | Suppression |
| **42** | Being obliged to keep up-to-date on new practices | Floor effect (≥ 50%); mean score <1.5 |  | Suppression |
| **43** | Inadequate space for downstream services |  | Clarity | Suppression |
| **44** | Competing with colleagues | Floor effect (≥ 50%); relevance <70%; redundancy | Clarity | Suppression |
| **45** | Taking care of a patient I know personally | Floor effect (≥ 50%) |  | Suppression |
| **46** | Decreased activity in the department | Floor effect (≥ 50%); relevance <70%; importance ("not at all" >20%) | Clarity | Suppression |
| **49** | Lack of user-friendliness space | Importance ("not at all" >20%) | Disturbance | Suppression |
| **50** | No consideration of the suffering experienced by patients’ families by other members of the healthcare team | Floor effect (≥ 50%) | Clarity | Suppression |
| **53** | Increased responsibilities associated with a change in status | Floor effect (≥ 50%); relevance <70%; redundancy | Clarity | Suppression |
| **56** | Uncertainty in the future of one’s career | Relevance <70% | Clarity | Suppression |
| **63** | Difficulties in seeking help from a colleague, seeking a physician | Floor effect (≥ 50%); mean score <1.5; importance ("not at all" >20%) |  | Suppression |
| **64** | Devaluation of skills because of one’s gender or family status | Floor effect (≥ 50%); relevance <70%; mean score <1.5; redundancy | Clarity | Suppression |
| **66** | Research takes precedence over patient management in the department | Floor effect (≥ 50%); relevance <70%; redundancy | Clarity; disturbance; other comment | Suppression |
| **71** | Economic and/or material constraints preventing optimal patient care | Relevance <70%; redundancy |  | Suppression |
| **72** | Stop treatment | Floor effect (≥ 50%); redundancy | Clarity; disturbance | Suppression |
| **74** | Staff changes or welcoming new healthcare professionals in the department | Floor effect (≥ 50%) |  | Suppression |
| **76** | Knowing how to prioritize care in emergency situations | Floor effect (≥ 50%) |  | Suppression |
| **77** | Being scorned when I am not doing research | Floor effect (≥ 50%); relevance <70%; importance ("not at all" >20%); redundancy | Clarity; disturbance | Suppression |
| **83** | Ambient temperature | Relevance <70% | Clarity; disturbance | Suppression |
| **84** | Taking care of a patient whose behavior or lifestyle have deteriorated his or her health | Importance ("not at all" >20%) |  | Suppression |
| **88** | Lack of a platform to express one's feeling at work | Floor effect (≥ 50%) | Disturbance | Suppression |
| **93** | Responsible for training and mentoring a colleague | Floor effect (≥ 50%) |  | Suppression |
| **94** | Presence of families during care | Floor effect (≥ 50%) | Other comment | Suppression |
| **95** | Complexity of the decision to stop or limit treatment |  | To discuss | Suppression |
| **96** | Having to constantly change units | Floor effect (≥ 50%); relevance <70%; importance ("not at all" >20%) | Disturbance | Suppression |
| **99** | Insufficient time provided by the supervisor | Floor effect (≥ 50%); redundancy | Clarity; disturbance | Suppression |

**Box 1 : Details of Statistical Analysis**

Participants with more than 5 missing values for the entire PS-ICU scale were excluded. First, we performed descriptive analyses of the general characteristics of each sample (frequency, means and standard deviations).

Then, to determine the best factor structure for the PS-ICU scale, we applied and compared the results from six variants of factor analyses, combining three different methods of extraction (principal component, principal axis and maximum likelihood) and two axis rotation techniques (Oblimin and Varimax). Preliminary, we examined the Kaiser-Meyer-Olkin (KMO) statistic and the result of the Bartlett’s test to check the sampling adequacy. To determine the number of factors to be extracted, we used and compared three criteria: the scree plot test of Catell (which suggested to extract one general stress factor or five factors), Horn’s parallel analysis (which suggested to extract six factors), the number of factors (i.e., eight) suggested by the systematic review of Laurent et al. (2020) [1] and the number of factors (i.e., seven) suggested by the qualitative interview analysis conducted in Phase 1. Thus, we compared the results obtained by the six factor analysis variants for the five-factor, six-factor, seven-factor and an eight-factor structures (Supplementary material – Table 3). The objective was to identify the factor solution that was the most invariant across the different extraction and rotation methods used. This strategy also aimed at identifying items with unstable factor loadings (i.e., those that not always load with the same other items or frequently having low loadings i.e., <0.30). We also identify items with frequent cross-loadings. Accordingly, the six-factor structure was the most invariant and four items (14^[[1]](#footnote-1)^, 22^[[2]](#footnote-2)^, 43^[[3]](#footnote-3)^, 47^[[4]](#footnote-4)^) frequently had low loadings (<.30) across techniques. The principal axis factoring method coupled with the Oblimin rotation technique obtained fewer cross loadings than other factor analysis variants. Thus, we decided to carry out a new analysis using this factor analysis variant, without the four problematic items and with a six-factor structure. We iteratively removed items that cross-load (i.e., that have a poor discriminant validity) *excepting* if 1) they have communalities above 0.30 and KMO values above .60 (i.e., explaining a fair proportion of shared variance, [2]), 2) if they loaded more on the general stress factor (> .50 on the first unrotated factor) and 3) if they were rated on average as amongst the most stressful by participants (i.e., above the sample mean stress score for the entire scale). Any item with loadings < 0.30 for all factors was also removed. After this step, items 30, 31, 39 was no longer considered due to their problematic loadings. Although items 14, 22, 30, 31, 39, 43, 47 were not included in the factor analyses, we decided to keep them in the calculation of the overall score (i.e., general factor) because of their relevance to the assessment of perceived stress by ICU professionals.

Item Response Theory (IRT) models were subsequently used to complement the results of factor analysis. We applied the Partial Credit Model (PCM), which is suitable for an ordinal responses scale [3]. An item difficulty parameter is estimated for each item on a logit scale. An item with a high difficulty parameter indicates that few patients choose a high response category to this item (considering this item as difficult to succeed). The PCM model was applied for each of the six dimensions identified by factor analysis. The adjustment of the model to the data was explored with global and individual item-fit statistics. Fit residual was also examined: a high positive residual (>2.5) indicated an unexpected response patterns, whereas a high negative residual indicated possible redundancy with other items.

To calculate the score for each factor, we tolerated less than 20% missing values per factor. The score of each factor was obtained by averaging the scores of items that loaded on it in factor analysis. Because the 50 items obtained loadings above .40 on the first unrotated general stress factor, we decided to use all of them to calculate the total PS-ICU score (mean score of all items). The higher the score, the higher the perceived stress intensity.

Then, to observe whether there were differences between men and women on the one hand, and according to the occupational status on the other hand, on the different scores on the PS-ICU scale, we performed ANOVAs with occupations or gender as independent variables, as between-subjects, and each PS-ICU score alternatively as dependent variable.

We next examined the internal consistency for each factor via Cronbach’s alphas and McDonald’s omega coefficients, and we examined the test-retest reliability using intraclass correlation coefficients for participants who experienced a significant event between the test and retest and those who did not (to estimate the sensitivity to change). The internal consistency was estimated for each factor, with all items given an equal weight. We considered Cronbach’s alphas < .70 as not satisfactory. Intraclass correlation coefficients were calculated between Time 1 and Time 2 PS-ICU scale scores, with statistics above .70 as a satisfactory. Because PS-ICU scores could depend on events experienced between the two measurement times, we conducted analyses with participants who had not reported significant event and, separately with participants who had reported significant event.

Finally, we used Pearson correlation coefficients and multiple regression analyses to examine the convergent/divergent validity of the PS-ICU scale with the four JCQ scores and the concurrent validity of the PS-ICU scale with the three scores of the MBI-HSS. In particular, multiple regression analyses estimated whether each stress factor measured by PS-ICU was associated with the three burnout dimensions, while controlling for the effects of the other five stress factors. If different patterns of results emerged for each PS-ICU stress factor, this would establish their discriminant predictive validity and, therefore, the relevance of their differentiation. The multicollinearity was checked, using VIF values below 4 and tolerance values greater than 0.2 as discarding any issue at this level [4].

The sample size was adequate for each statistical used. For exploratory factor analysis, a sample size of at least 300 participants generally provides replicable factors [5], with a sample size around 500 participants considered as “very good” [6]. Concerning IRT, a sample of 200 to 500 participants is most frequently recommended to obtain accurate and stable statistics [3]. A sample size of 320 participants produces a two-sided 95% confidence interval with a width equal to 0.20 for a Pearson's correlation coefficient of at least 0.30 [7]. For as scale containing at least 5 items, a sample of 348 participants is enough to estimate a coefficient alpha of at least 0.70 with a two-sided 95% confidence interval and a width of 0.10 [8]. The estimation of an intraclass correlation coefficient of at least 0.80 with a two-sided 95% confidence interval and a width of 0.10 necessitates a sample of at least 204 participants [9].

Statistical analyses were carried out with SPSS version 26.0, Jamovi 1.1.5 (Retrieved from https://www.jamovi.org), SAS software (version 9.4), RUMM2020 and PASS2020. The *p*-value for statistical significance was set at *p* <.05.

**Table 3.**

Comparisons between different methods of extraction (principal component, principal axis and maximum likelihood) and rotation techniques (Oblimin and Varimax) with six-factor structure and without items 22, 14, 43, 47.

| Items | | Without items 22 – 14 – 43 – 47 | | | | | | | | | | | | |
| --- | --- | --- | --- | --- | --- | --- | --- | --- | --- | --- | --- | --- | --- | --- |
| Rotation | | Oblimin | | | |  | Varimax | | | | | | | |
| Method | | Maximum likelihood | Principal axis | | Principal component |  | Maximum likelihood | | Principal axis | Principal component | | | | |
| 6 | Shortage of beds in the unit | 1 | 1 | | 1 |  | 1 | | 1 | 1 | | | | |
| 9 | Disagreement and/or lack of coordination with other units concerning a patient’s treatment | 1 | 1 | | 1 |  | 1 | | 1 | 1 | | | | |
| 10 | Family conflict or disagreement concerning the patient’s treatment plan | 1 | 1 | | 1 |  | 1 | | 1 | 1 | | | | |
| 35 | Family which does not trust me or does not trust the team | 1 | 1 | | 1 |  | 1 | | 1 | 1 | | | | |
| 41 | Caring for a patient who should not be treated by the ICU | 1 | 1 | | 1 |  | 1 | | 1 | 1 | | | | |
| 42 | Uncertainty concerning the diagnosis or the therapy project of the patient | 1 | 1 | | 1 |  | 1 | | 1 | 1 | | | | |
| 4* | Contradictory information given by other healthcare professionals to the family | 1 | 1 | | 1 |  | 1 | | 1 | 1_4 | | | | |
| 15 | Lack of support from the administration | 1 | 1 | | 1 |  | 1 | | 1 | 1_5 | | | | |
| 26 | Family whose beliefs or lifestyle are contradictory with my values or the functioning of the unit | 1 | 1 | | 1 |  | 1_2 | | 1_2 | 1 | | | | |
| 27 | Family’s misunderstanding of the gravity of the diagnosis or the prognosis of the patient | 1 | 1 | | 1 |  | 1_2 | | 1_2 | 1_2 | | | | |
| 39* | Time spent on administrative tasks at the expense of care giving time | 1 |  | |  |  | 1 | | 1_5 | 4_1 | | | | |
| 1 | Socially isolated end-of-life patient or one with no immediate family | 2 | 2 | | 2 |  | 2 | | 2 | 2 | | | | |
| 28 | Series of patient deaths in the unit over a short period | 2 | 2 | | 2 |  | 2 | | 2 | 2 | | | | |
| 29 | Patient who makes me think of someone close to me or of myself | 2 | 2 | | 2 |  | 2 | | 2 | 2 | | | | |
| 34 | Death of a patient with whom I had developed special ties | 2 | 2 | | 2 |  | 2 | | 2 | 2 | | | | |
| 45* | Having to announce a bad diagnosis to the patient or his/her family or be present when such a diagnosis is announced | 2 | 2 | | 2 |  | 2 | | 2 | 2 | | | | |
| 44* | Patient suffering physically or psychologically | 2 | 2 | | 2 |  | 2_1 | | 2_1 | 2_1 | | | | |
| 49 | Decision to stop or reduce treatment | 2 | 2 | | 2_1 |  | 2_1 | | 2_1 | 2_1 | | | | |
| 5 | Caring for young patients or who have young children | 2 | 2 | | 2_3 |  | 2_3 | | 2_3 | 2_3 | | | | |
| 7* | Families’ distress or emotions | 2 | 2 | | 2_3 |  | 2_3 | | 2_3 | 2_3_5 | | | | |
| 30 | Powerlessness or incompetence in supporting families | 2 |  | | 2 |  | 2 | | 2 | 2_1 | | | | |
| 20 | Working pace or working hours hardly compatible with family or social life | 3 | 4 | | 4 |  | 4 | | 4 | 6 | | | | |
| 23 | Schedule change, overtime | 3 | 4 | | 4 |  | 4 | | 4 | 6 | | | | |
| 50* | Being on call or working nights | 3 | 4 | | 4 |  | 4 | | 4 | 6 | | | | |
| 36* | Continuous and heavy workload | 3 | 4 | | 4 |  | 4 | | 4 | 6_5 | | | | |
| 46 | Lack of equality in the distribution of tasks among healthcare professionals | 3 | 4 | | 4 |  | 1_4 | | 4 | 6_5_4 | | | | |
| 16 | Risk of error, fear of doing a poor job | 4 | 3 | | 3 |  | 3 | | 3 | 3 | | | | |
| 18 | Having to execute care tasks quickly in emergency cases | 4 | 3 | | 3 |  | 3 | | 3 | 3 | | | | |
| 37 | Having to perform tasks for which I have neither knowledge nor skills | 4 | 3 | | 3 |  | 3 | | 3 | 3 | | | | |
| 40 | Treating complex or serious pathologies | 4 | 3 | | 3 |  | 3 | | 3 | 3 | | | | |
| 25* | Patient who deteriorates in an unexpected or unexplained manner | 4 | 3 | | 3 |  | 3_2 | | 3_2 | 3_2_1 | | | | |
| 13 | Difficulty to find my place, have my skills recognized, or voice my opinion within the team | 5 | 5 | | 5 |  | 5 | | 5 | 4 | | | | |
| 17* | Negative atmosphere prevailing in the team, gossip, rumours within the team | 5 | 5 | | 5 |  | 5 | | 5 | 4 | | | | |
| 3* | Lack of recognition (from the patient, the family, the team, the hierarchy) | 5 | 5 | | 5 |  | 5_1 | | 5 | 4 | | | | |
| 21 | Conflicts with members of the healthcare team | 5 | 5_1 | | 5 |  | 5_1 | | 5_1 | 4_1 | | | | |
| 8 | Inadequate or under-equipped healthcare space or defective materials | 6 | 6 | | 6 |  | 6_1 | | 6 | 5 | | | | |
| 2* | Colleague not doing his/her work properly | 6 | 6 | | 6 |  | 6_1_5 | | 6_5_1 | 5_4 | | | | |
| 12* | Incomprehensible or unnecessary care relative to the patient’s situation | 6 | 6 | | 6 |  | 6_1_5 | | 6_5_1 | 5_4 | | | | |
| 11* | Too many professionals around the patient in an emergency situation | 6 | 6 | | 6_3 |  | 6_3_5 | | 6_3_5 | 5_3_4 | | | | |
| 48* | Accumulated workloads resulting from clinical activity, training, research or teaching | 3_1 | 4_1 | | 4_1 |  | 4_1 | | 4_1 | 6_1_4 | | | | |
| 24 | Working while experiencing difficult personal events | 3_2 | 4_2 | | 4_2 |  | 2_4 | | 2_4 | 6_2 | | | | |
| 19 | Plaintive patient who makes many requests | 4_6 | 3_6 | | 6_3 |  | 6_3 | | 6_3 | 5_3 | | | | |
| 38 | Assessed or judged by the other members of the team | 5_4 | 5_3 | | 5_3 |  | 5_3 | | 5_3 | 3_4 | | | | |
| 32* | Lack of staff | 6_3 | 4_6 | | 6_4 |  | 6_4 | | 6_4 | 5_6 | | | | |
| 33 | Non-supportive, aggressive or delirious patient | 6_4 | 6 | | 6 |  | 6_3 | | 6 | 5 | | | | |
| 31* | Changes in the modalities of care or the therapy project depending on the doctor responsible for the patient |  |  | |  |  | 1 | | 1 | 1_4_5 | | | | |
| 14 | Noisy environment |  |  | |  |  |  | |  |  | | | | |
| 22 | Not being able to communicate with the patient |  |  | |  |  |  | |  |  | | | | |
| 43 | Lack of respect for the patient (against his/her wishes, his/her integrity, his/her situation, etc.) |  | | | | | |  | | | | | | |
| 47 | Unsuitable or under-equipped space to receive families | | |  | | | | | |  |  |  |  |  |

*Note.* Les items considérés comme importants (i.e., ceux qui saturent le plus et qui ont un score moyen élevé) sont notés par une *.

**Table 4**

Difficulties and fit statistics for the PS-ICU items from Item Response Theory models

|  | Difficulty parameter | Standard Error | Fit residual | Chi2 | p-value | p-value | PSI* |
| --- | --- | --- | --- | --- | --- | --- | --- |
| *Factor 1* |  |  |  |  |  | <0.01 | 0.83 |
| Item 4 | -0.406 | 0.052 | -0.969 | 9.337 | 0.23 |  |  |
| Item 6 | -0.15 | 0.048 | 2.990 | 23.189 | 0.002 |  |  |
| Item 9 | -0.239 | 0.05 | -0.037 | 19.076 | 0.008 |  |  |
| Item 10 | 0.111 | 0.051 | 0.367 | 9.633 | 0.21 |  |  |
| Item 15 | -0.027 | 0.043 | 2.656 | 15.618 | 0.029 |  |  |
| Item 26 | 0.535 | 0.056 | -0.288 | 7.078 | 0.421 |  |  |
| Item 27 | -0.075 | 0.06 | -0.791 | 14.333 | 0.046 |  |  |
| Item 35 | 0.302 | 0.046 | 1.683 | 30.911 | <0.001 |  |  |
| Item 41 | 0.004 | 0.057 | 0.099 | 12.188 | 0.095 |  |  |
| Item 42 | -0.054 | 0.059 | -0.073 | 10.449 | 0.165 |  |  |
| *Factor 2* |  |  |  |  |  | 0.04 | 0.83 |
| Item 1 | 0.471 | 0.061 | 0.940 | 14.340 | 0.045 |  |  |
| Item 5 | -0.487 | 0.053 | 1.853 | 8.349 | 0.303 |  |  |
| Item 7 | -0.956 | 0.061 | -0.510 | 11.576 | 0.115 |  |  |
| Item 28 | 0.437 | 0.055 | -0.088 | 5.281 | 0.626 |  |  |
| Item 29 | 0.252 | 0.047 | 1.908 | 7.988 | 0.342 |  |  |
| Item 34 | 0.504 | 0.046 | 0.301 | 9.818 | 0.199 |  |  |
| Item 44 | -0.629 | 0.058 | 0.256 | 11.951 | 0.102 |  |  |
| Item 45 | -0.172 | 0.057 | -1.185 | 11.574 | 0.115 |  |  |
| Item 49 | 0.581 | 0.057 | -0.140 | 2.804 | 0.902 |  |  |
| *Factor 3* |  |  |  |  |  | 0.07 | 0.77 |
| Item 16 | -0.26 | 0.055 | -1.548 | 14.245 | 0.047 |  |  |
| Item 18 | 0.06 | 0.061 | -0.612 | 9.604 | 0.212 |  |  |
| Item 25 | -0.182 | 0.062 | 2.936 | 11.823 | 0.107 |  |  |
| Item 37 | 0.226 | 0.048 | 1.135 | 5.003 | 0.660 |  |  |
| Item 40 | 0.156 | 0.065 | -0.319 | 7.507 | 0.378 |  |  |
| *Factor 4* |  |  |  |  |  | 0.003 | 0.8 |
| Item 20 | -0.327 | 0.05 | -1.465 | 15.52 | 0.0298 |  |  |
| Item 23 | 0.087 | 0.052 | -0.435 | 12.507 | 0.085 |  |  |
| Item 24 | 0.235 | 0.044 | 3.567 | 11.413 | 0.121 |  |  |
| Item 32 | -0.134 | 0.048 | 1.460 | 4.877 | 0.675 |  |  |
| Item 36 | -0.255 | 0.051 | -2.645 | 25.36 | <0.001 |  |  |
| Item 46 | 0.35 | 0.047 | 2.245 | 10.52 | 0.161 |  |  |
| Item 48 | 0.186 | 0.046 | 1.097 | 7.934 | 0.338 |  |  |
| Item 50 | -0.142 | 0.05 | 1.328 | 1.423 | 0.984 |  |  |
| *Factor 5* |  |  |  |  |  | 0.31 | 0.72 |
| Item 3 | -0.325 | 0.05 | 0.723 | 7.595 | 0.37 |  |  |
| Item 13 | 0.323 | 0.045 | -0.671 | 8.793 | 0.268 |  |  |
| Item 17 | -0.13 | 0.046 | -0.759 | 11.242 | 0.128 |  |  |
| Item 21 | 0.135 | 0.046 | 1.328 | 2.872 | 0.897 |  |  |
| Item 38 | -0.003 | 0.048 | 1.995 | 8.062 | 0.327 |  |  |
| *Factor 6* |  |  |  |  |  | 0.43 | 0.76 |
| Item 2 | -0.461 | 0.056 | -0.161 | 2.553 | 0.923 |  |  |
| Item 8 | 0.172 | 0.047 | 0.344 | 8.011 | 0.330 |  |  |
| Item 11 | 0.005 | 0.053 | 1.225 | 4.742 | 0.690 |  |  |
| Item 12 | 0.107 | 0.05 | 0.078 | 14.039 | 0.050 |  |  |
| Item 19 | 0.387 | 0.062 | -0.386 | 7.253 | 0.400 |  |  |
| Item 33 | -0.211 | 0.056 | 0.822 | 6.250 | 0.510 |  |  |

*Note.* *PSI: Person separation index

Table 5. Mean comparisons (ANOVAs) according to between-subjects (occupation and gender) and within-subjects (stress factors) variables.

|  | | Stress factors | | | | | | |
| --- | --- | --- | --- | --- | --- | --- | --- | --- |
|  |  | Factor 1. Families-related and organizational difficulties | Factor 2. Patient- and family-related emotional load | Factor 3. Patient's critical condition and risk/skill-related issues | Factor 4. Quantitative load and human-resources management issues | Factor 5. Work-team-related difficulties | Factor 6. Suboptimal care and related causes | Factor G. Overall stress exposure |
| Between-subject comparisons | |  |  |  |  |  |  |  |
| Model *R*² | | .16 | .11 | .13 | .07 | .10 | .13 | .10 |
| Occupation | *F*(2) | 12.55*** | 0.11 | 3.71 | 0.29 | 3.93* | 42.73*** | 0.66 |
|  | Physicians | 2.13 | 2.1 | 2.23 | 2.29 | 2.20 | 2.16 | 2.18 |
|  | Nurses | 1.92 | 2.12 | 2.37 | 2.33 | 2.03 | 2.58 | 2.23 |
| Gender | *F*(1) | 0.90 | 24.97*** | 13.66*** | 5.41* | 5.02* | 1.09 | 9.42*** |
|  | Men | 2.08 | 1.90 | 2.11 | 2.12 | 2.16 | 2.40 | 2.11 |
|  | Women | 2.15 | 2.26 | 2.43 | 2.32 | 2.38 | 2.48 | 2.30 |
| Within-subject comparisons  (stress factors):  Pillai’s trace (5) = .07*** | | 2.12 _cb_ | 2.08 _cb_ | 2.27 _ab_ | 2.22 _ab_ | 2.27 _ab_ | 2.44 _a_ | 2.20 |

*Note*. For all stress factors the equality of error variances is assumed (non-significant Levene’s tests). * *p* < .05, ** *p* < .01, *** *p* < .001. When the omnibus effect was significant for between-subject variables with more than two groups, Bonferroni’s adjusted pairwise comparison test was used. Means having dissimilar subscripts (_a b c_) differ significantly according to this test (*p* < .05).

**Box 2 : PS-ICU scale French, English, Italian, Spanish version**

**PS-ICU scale English version**

**Perceived stressors in intensive care units**

Below are several situations that may be experienced in intensive care units. For each of these situations: Indicate whether or not you have experienced a specific situation within your unit and, if so, specify your level of stress when you encountered the situation. Respond by circling one number ranging from 0 (never experienced) to 4 (experienced and extremely stressed).

| **Have you experienced this situation within your unit?** | **Never experienced** | **I experienced this situation and...** | | | | |
| --- | --- | --- | --- | --- | --- | --- |
|  |  | **I was not at all stressed** | **I was a little stressed** | **I was rather stressed** | **I was extremely stressed** | |
| 1. Socially isolated end-of-life patient or one with no immediate family | 0 | 1 | 2 | 3 | 4 | |
| 1. Colleague not doing his/her work properly | 0 | 1 | 2 | 3 | 4 | |
| 1. Lack of recognition (from the patient, the family, the team, the hierarchy) | 0 | 1 | 2 | 3 | 4 | |
| 1. Contradictory information given by other healthcare professionals to the family | 0 | 1 | 2 | 3 | 4 | |
| 1. Caring for young patients or who have young children | 0 | 1 | 2 | 3 | 4 | |
| 1. Shortage of beds in the unit | 0 | 1 | 2 | 3 | 4 | |
| 1. Families’ distress or emotions | 0 | 1 | 2 | 3 | 4 | |
| 1. Inadequate or under-equipped healthcare space or defective materials | 0 | 1 | 2 | 3 | 4 | |
| 1. Disagreement and/or lack of coordination with other units concerning a patient’s treatment | 0 | 1 | 2 | 3 | 4 | |
| 1. Family conflict or disagreement concerning the patient’s treatment plan | 0 | 1 | 2 | 3 | 4 | |
| 1. Too many professionals around the patient in an emergency situation | 0 | 1 | 2 | 3 | 4 | |
| 1. Incomprehensible or unnecessary care relative to the patient’s situation | 0 | 1 | 2 | 3 | 4 | |
| 1. Difficulty to find my place, have my skills recognized, or voice my opinion within the team | 0 | 1 | 2 | 3 | 4 | |
| 1. Noisy environment | 0 | 1 | 2 | 3 | 4 | |
| 1. Lack of support from the administration | 0 | 1 | 2 | 3 | 4 | |
| 1. Risk of error, fear of doing a poor job | 0 | 1 | 2 | 3 | 4 | |
| 1. Negative atmosphere prevailing in the team, gossip, rumours within the team | 0 | 1 | 2 | 3 | 4 | |
| 1. Having to execute care tasks quickly in emergency cases | 0 | 1 | 2 | 3 | 4 | |
| 1. Plaintive patient who makes many requests | 0 | 1 | 2 | 3 | 4 | |
| 1. Working pace or working hours hardly compatible with family or social life | 0 | 1 | 2 | 3 | 4 | |
| 1. Conflicts with members of the healthcare team | 0 | 1 | 2 | 3 | 4 | |
| 1. Not being able to communicate with the patient | 0 | 1 | 2 | 3 | 4 | |
| 1. Schedule changes, overtime | 0 | 1 | 2 | 3 | | 4 |
| 24. Working while experiencing difficult personal events | 0 | 1 | 2 | 3 | | 4 |
| 1. Patient who deteriorates in an unexpected or unexplained manner | 0 | 1 | 2 | 3 | | 4 |
| 1. Family whose beliefs or lifestyle are contradictory with my values or the functioning of the unit | 0 | 1 | 2 | 3 | | 4 |
| 1. Family’s misunderstanding of the gravity of the diagnosis or the prognosis of the patient | 0 | 1 | 2 | 3 | | 4 |
| 1. Series of patient deaths in the unit over a short period | 0 | 1 | 2 | 3 | | 4 |
| 1. Patient who makes me think of someone close to me or of myself | 0 | 1 | 2 | 3 | | 4 |
| 1. Powerlessness or incompetence in supporting families | 0 | 1 | 2 | 3 | | 4 |
| 1. Changes in the modalities of care or the therapy project depending on the doctor responsible for the patient | 0 | 1 | 2 | 3 | | 4 |
| 1. Lack of staff | 0 | 1 | 2 | 3 | | 4 |
| 1. Non-supportive, aggressive or delirious patient | 0 | 1 | 2 | 3 | | 4 |
| 1. Death of a patient with whom I had developed special ties | 0 | 1 | 2 | 3 | | 4 |
| 1. Family which does not trust me or does not trust the team | 0 | 1 | 2 | 3 | | 4 |
| 1. Continuous and heavy workload | 0 | 1 | 2 | 3 | | 4 |
| 1. Having to perform tasks for which I have neither knowledge nor skills | 0 | 1 | 2 | 3 | | 4 |
| 1. Assessed or judged by the other members of the team | 0 | 1 | 2 | 3 | | 4 |
| 1. Time spent on administrative tasks at the expense of care giving time | 0 | 1 | 2 | 3 | | 4 |
| 1. Treating complex or serious pathologies | 0 | 1 | 2 | 3 | | 4 |
| 1. Caring for a patient who should not be treated by the ICU | 0 | 1 | 2 | 3 | | 4 |
| 1. Uncertainty concerning the diagnosis or the therapy project of the patient | 0 | 1 | 2 | 3 | | 4 |
| 1. Lack of respect for the patient (against his/her wishes, his/her integrity, his/her situation, etc.) | 0 | 1 | 2 | 3 | | 4 |
| 1. Patient suffering physically or psychologically | 0 | 1 | 2 | 3 | | 4 |
| 1. Having to announce a bad diagnosis to the patient or his/her family or be present when such a diagnosis is announced | 0 | 1 | 2 | 3 | | 4 |
| 1. Lack of equality in the distribution of tasks among healthcare professionals | 0 | 1 | 2 | 3 | | 4 |
| 1. Unsuitable or under-equipped space to receive families | 0 | 1 | 2 | 3 | | 4 |
| 1. Accumulated workloads resulting from clinical activity, training, research or teaching | 0 | 1 | 2 | 3 | | 4 |
| 1. Decision to stop or reduce treatment | 0 | 1 | 2 | 3 | | 4 |
| 1. Being on call or working nights | 0 | 1 | 2 | 3 | | 4 |

**PS-ICU scale French version**

**Stress perçu en réanimation**

Vous trouverez ci-dessous plusieurs situations professionnelles pouvant être vécues en réanimation. Pour chacune de ces situations : indiquez si vous l’avez vécue au sein de votre service et, si oui, précisez à quel point vous avez ressenti du stress face à cette situation. Pour répondre, entourez un chiffre allant de 0 (non vécu(e)) à 4 (vécu(e) et très stressé(e)).

| **Avez-vous vécu cette situation au sein de votre service ?** | **Non vécu(e)** | **J’ai vécu cette situation et…** | | | | | |
| --- | --- | --- | --- | --- | --- | --- | --- |
|  |  | **je n’ai pas du tout été stressé(e)** | **j’ai été  un peu stressé(e)** | **j’ai été assez stressé(e)** | **j’ai été très stressé(e)** | | |
| 1. Patient en fin de vie isolé socialement ou familialement | 0 | 1 | 2 | 3 | 4 | | |
| 2. Collègue qui ne fait pas correctement son travail | 0 | 1 | 2 | 3 | 4 | | |
| 3. Manque de reconnaissance (de la part du patient, de la famille, de l’équipe, la hiérarchie) | 0 | 1 | 2 | 3 | 4 | | |
| 4. Informations contradictoires données par d'autres soignants à la famille | 0 | 1 | 2 | 3 | 4 | | |
| 5. Prise en charge de patients jeunes ou ayant des enfants en bas âge | 0 | 1 | 2 | 3 | 4 | | |
| 6. Manque de lits dans le service | 0 | 1 | 2 | 3 | 4 | | |
| 7. Détresse ou émotions des familles | 0 | 1 | 2 | 3 | 4 | | |
| 8. Espace de soin inadapté, sous-équipé ou matériel défectueux | 0 | 1 | 2 | 3 | 4 | | |
| 9. Désaccord ou manque de coordination avec les autres services sur la prise en charge du patient | 0 | 1 | 2 | 3 | 4 | | |
| 10. Conflit ou désaccord au sein de la famille concernant la prise en charge du patient | 0 | 1 | 2 | 3 | 4 | | |
| 11. Afflux trop important de professionnels autour du malade dans une situation d’urgence | 0 | 1 | 2 | 3 | 4 | | |
| 12. Réaliser des soins incompréhensibles ou inutiles au regard de la situation du patient | 0 | 1 | 2 | 3 | 4 | | |
| 13. Difficulté à faire ma place, à faire reconnaître mes compétences ou faire entendre mon avis au sein de l'équipe | 0 | 1 | 2 | 3 | 4 | | |
| 14. Environnement bruyant | 0 | 1 | 2 | 3 | 4 | | |
| 15. Absence de soutien de l'administration | 0 | 1 | 2 | 3 | 4 | | |
| 16. Risque d'erreur, peur de mal faire son travail | 0 | 1 | 2 | 3 | 4 | | |
| 17. Mauvaise ambiance dans l'équipe, commérages, rumeurs au sein de l'équipe | 0 | 1 | 2 | 3 | 4 | | |
| 18. Devoir exécuter rapidement des soins en situation d'urgence | 0 | 1 | 2 | 3 | 4 | | |
| 19. Patient plaintif, exprimant de nombreuses demandes | 0 | 1 | 2 | 3 | 4 | | |
| 20. Rythme ou horaire de travail difficilement compatible avec la vie de famille ou la vie sociale | 0 | 1 | 2 | 3 | 4 | | |
| 21. Conflits avec des membres de l'équipe soignante | 0 | 1 | 2 | 3 | 4 | | |
| 22. Ne pas pouvoir communiquer avec le patient | 0 | 1 | 2 | 3 | 4 | | |
| 23. Changement de planning, heures supplémentaires | 0 | 1 | 2 | 3 | | 4 |  |
| 24. Travailler alors que l'on vit des événements personnels difficiles | 0 | 1 | 2 | 3 | | 4 |  |
| 25. Patient qui se dégrade de manière inattendue et inexpliquée | 0 | 1 | 2 | 3 | | 4 |  |
| 26. Famille dont les croyances ou le mode de vie sont en contradiction avec mes valeurs ou le mode de fonctionnement du service | 0 | 1 | 2 | 3 | | 4 |  |
| 27. Mauvaise compréhension par la famille de la gravité du diagnostic ou du pronostic du patient | 0 | 1 | 2 | 3 | | 4 |  |
| 28. Enchainement de décès de patients dans le service sur une période courte | 0 | 1 | 2 | 3 | | 4 |  |
| 29. Patient qui me fait penser à un de mes proches ou à moi-même | 0 | 1 | 2 | 3 | | 4 |  |
| 30. Impuissance ou incompétence dans l'accompagnement des familles | 0 | 1 | 2 | 3 | | 4 |  |
| 31. Changement des modalités de soins ou du projet thérapeutique en fonction du médecin responsable du patient | 0 | 1 | 2 | 3 | | 4 |  |
| 32.  Manque de personnel | 0 | 1 | 2 | 3 | | 4 |  |
| 33.  Patient non conciliant, agressif ou délirant | 0 | 1 | 2 | 3 | | 4 |  |
| 34.  Mort d'un patient avec lequel j'avais créé des liens particuliers | 0 | 1 | 2 | 3 | | 4 |  |
| 35.  Famille qui ne me fait pas confiance ou qui ne fait pas confiance à l'équipe | 0 | 1 | 2 | 3 | | 4 |  |
| 36.  Charge de travail importante et permanente | 0 | 1 | 2 | 3 | | 4 |  |
| 37.  Devoir effectuer des tâches pour lesquelles je manque de connaissances ou de compétences | 0 | 1 | 2 | 3 | | 4 |  |
| 38.  Être évalué ou jugé par les autres membres de l'équipe | 0 | 1 | 2 | 3 | | 4 |  |
| 39.  Temps consacré à des tâches administratives au détriment du temps de soins | 0 | 1 | 2 | 3 | | 4 |  |
| 40.  Prise en charge de pathologies graves ou complexes | 0 | 1 | 2 | 3 | | 4 |  |
| 41.  M'occuper d'un patient dont les soins ne relèvent pas de la réanimation | 0 | 1 | 2 | 3 | | 4 |  |
| 42.  Incertitude du diagnostic ou du projet thérapeutique du patient | 0 | 1 | 2 | 3 | | 4 |  |
| 43.  Manque de respect vis-à-vis du patient (vis-à-vis de ses volontés, de son intégrité, sa situation, etc.) | 0 | 1 | 2 | 3 | | 4 |  |
| 44.  Patient qui souffre physiquement ou psychiquement | 0 | 1 | 2 | 3 | | 4 |  |
| 45.  Devoir annoncer au patient ou à la famille un diagnostic de maladie grâve ou être présent lors de l’annonce d’un tel diagnostic | 0 | 1 | 2 | 3 | | 4 |  |
| 46.  Manque d’égalité dans la répartition du travail entre professionnels | 0 | 1 | 2 | 3 | | 4 |  |
| 47.  Espace d'accueil des familles inadapté ou sous-équipé | 0 | 1 | 2 | 3 | | 4 |  |
| 48.  Cumul de la charge de travail liée à l’activité clinique, la formation, la recherche ou l’enseignement | 0 | 1 | 2 | 3 | | 4 |  |
| 49.  Prise de décisions d’arrêt ou limitation de traitement | 0 | 1 | 2 | 3 | | 4 |  |
| 50.  Gardes ou travail de nuit | 0 | 1 | 2 | 3 | | 4 |  |

**PS-ICU scale Spanish version**

**Estresores percibidos en la unidad de reanimación**

A continuación, se presentan varias situaciones profesionales que pueden vivirse en reanimación. Para cada una de estas situaciones: indique si alguna vez las ha vivido en su servicio y, si es así, especifique hasta qué punto ha sentido estrés frente a dicha situación. Para responder, rodee con un círculo un número de 0 (nunca la he vivido) hasta 4 (he vivido esta situación y he sentido mucho estrés).

| **¿Ha vivido alguna vez esta situación en su servicio?** | **Nunca la he vivido** | **He vivido esta situación y…** | | | |
| --- | --- | --- | --- | --- | --- |
|  |  | **no he sentido nada de estrés** | **he sentido un poco de estrés** | **he sentido bastante estrés** | **he sentido mucho estrés** |
| 1. Paciente en etapa final de su vida aislado social o familiarmente | 0 | 1 | 2 | 3 | 4 |
| 2. Compañero/a que no realiza correctamente su trabajo | 0 | 1 | 2 | 3 | 4 |
| 3. Falta de reconocimiento (de parte del paciente, la familia, el equipo o la jerarquía) | 0 | 1 | 2 | 3 | 4 |
| 4. Informaciones contradictorias dadas a la familia por parte de otros cuidadores | 0 | 1 | 2 | 3 | 4 |
| 5. Tratamiento de pacientes jóvenes o que tienen niños pequeños | 0 | 1 | 2 | 3 | 4 |
| 6. Falta de camas en el servicio | 0 | 1 | 2 | 3 | 4 |
| 7. Desamparo o emociones de las familias | 0 | 1 | 2 | 3 | 4 |
| 8. Espacio de cuidados inadaptado, sin equipamiento suficiente o material defectuoso | 0 | 1 | 2 | 3 | 4 |
| 9. Desacuerdo o falta de coordinación con los demás servicios sobre el tratamiento del paciente | 0 | 1 | 2 | 3 | 4 |
| 10. Conflicto o desacuerdo entre los miembros de la familia sobre el tratamiento del paciente | 0 | 1 | 2 | 3 | 4 |
| 11. Afluencia demasiado importante de profesionales alrededor del paciente en una situación de emergencia | 0 | 1 | 2 | 3 | 4 |
| 12. Realizar cuidados incomprensibles o inútiles con respecto a la situación del paciente | 0 | 1 | 2 | 3 | 4 |
| 13. Dificultad para hacerse un sitio, para que reconozcan mis competencias o para que escuchen mi opinión dentro del equipo | 0 | 1 | 2 | 3 | 4 |
| 14. Entorno ruidoso | 0 | 1 | 2 | 3 | 4 |
| 15. Ausencia de apoyo por parte de la administración | 0 | 1 | 2 | 3 | 4 |
| 16. Riesgo de error, miedo a hacer mal mi trabajo | 0 | 1 | 2 | 3 | 4 |
| 17. Mal ambiente en el equipo, chismes, rumores dentro del equipo | 0 | 1 | 2 | 3 | 4 |
| 18. Tener que ejecutar rápidamente cuidados en situación de emergencia | 0 | 1 | 2 | 3 | 4 |
| 19. Paciente que se queja y pide muchas cosas | 0 | 1 | 2 | 3 | 4 |
| 20. Ritmo u horario de trabajo difícil de compatibilizar con la vida familiar o la vida social | 0 | 1 | 2 | 3 | 4 |
| 21. Conflictos con miembros del equipo médico y sanitario | 0 | 1 | 2 | 3 | 4 |
| 22. No poder comunicar con el paciente | 0 | 1 | 2 | 3 | 4 |
| 23. Cambios en la planificación, horas extras | 0 | 1 | 2 | 3 | 4 |
| 24. Trabajar mientras vivo acontecimientos personales difíciles | 0 | 1 | 2 | 3 | 4 |
| 25. Paciente que se degrada de manera inesperada e inexplicada | 0 | 1 | 2 | 3 | 4 |
| 25. Paciente que se degrada de manera inesperada e inexplicada | 0 | 1 | 2 | 3 | 4 |
| 26. Familia cuyas creencias o estilo de vida son contradictorios con mis valores o el modo de funcionamiento del servicio | 0 | 1 | 2 | 3 | 4 |
| 27. Mala comprensión por parte de la familia sobre la gravedad del diagnóstico o del pronóstico del paciente | 0 | 1 | 2 | 3 | 4 |
| 28. Cadena de fallecimientos de pacientes en el servicio en un periodo corto | 0 | 1 | 2 | 3 | 4 |
| 29. Paciente que me recuerda a uno de mis familiares o a mí mismo/a | 0 | 1 | 2 | 3 | 4 |
| 30. Impotencia o incompetencia en el acompañamiento de las familias | 0 | 1 | 2 | 3 | 4 |
| 31. Cambio de las modalidades de cuidados o del proyecto terapéutico en función del médico responsable del paciente | 0 | 1 | 2 | 3 | 4 |
| 32. Falta de personal | 0 | 1 | 2 | 3 | 4 |
| 33. Paciente que no colabora, agresivo o delirante | 0 | 1 | 2 | 3 | 4 |
| 34. Muerte de un paciente con el cual había creado vínculos especiales | 0 | 1 | 2 | 3 | 4 |
| 35. Familia que no confía en mí o que no confía en el equipo | 0 | 1 | 2 | 3 | 4 |
| 36. Carga de trabajo significativa y permanente | 0 | 1 | 2 | 3 | 4 |
| 37. Tener que efectuar tareas para las cuáles me faltan conocimientos o competencias | 0 | 1 | 2 | 3 | 4 |
| 38. Ser evaluado o juzgado por los otros miembros del equipo | 0 | 1 | 2 | 3 | 4 |
| 39. Tiempo dedicado a tareas administrativas en detrimento del tiempo de cuidados | 0 | 1 | 2 | 3 | 4 |
| 40. Tratamiento de patologías graves o complejas | 0 | 1 | 2 | 3 | 4 |
| 41. Ocuparme de un paciente cuyos cuidados no entran en el ámbito de la reanimación | 0 | 1 | 2 | 3 | 4 |
| 42. Incertidumbre sobre el diagnóstico o el proyecto terapéutico del paciente | 0 | 1 | 2 | 3 | 4 |
| 43. Falta de respeto hacia el paciente (sobre sus voluntades, su integridad, su situación, etc.) | 0 | 1 | 2 | 3 | 4 |
| 44. Paciente que sufre física o psíquicamente | 0 | 1 | 2 | 3 | 4 |
| 45. Tener que comunicar al paciente o a la familia un diagnóstico desfavorable o estar presente cuando se comunica un diagnóstico desfavorable | 0 | 1 | 2 | 3 | 4 |
| 46. Falta de igualdad en la distribución del trabajo entre profesionales | 0 | 1 | 2 | 3 | 4 |
| 47. Espacio de acogida de las familias inadaptado o sin equipamiento suficiente | 0 | 1 | 2 | 3 | 4 |
| 48. Acumulación de la carga de trabajo relacionada con la actividad clínica, la formación, la investigación o la enseñanza | 0 | 1 | 2 | 3 | 4 |
| 49. Toma de decisiones sobre la interrupción o la limitación del tratamiento | 0 | 1 | 2 | 3 | 4 |
| 50. Guardias o trabajo de noche | 0 | 1 | 2 | 3 | 4 |

**PS-ICU scale Italian version**

**Fattori percepiti come fonti di stress nelle unità di Terapia intensiva**

Elencate qui di seguito, troverà differenti situazioni professionali che possono essere vissute in un reparto di Terapia intensiva. Per ognuna di esse, indichi se ha già dovuto confrontarsi con questa situazione nel Suo reparto e, in tal caso, precisi in che misura si è sentito stressato a causa di questa situazione. Per rispondere, assegni una cifra da 0 (non ho mai dovuto confrontarmi a questa situazione) a 4 (mi sono già trovato/a in questa situazione e mi ha molto stressato/a).

| **Ha già dovuto confrontarsi con questa situazione nel Suo reparto?** | | **No, mai** | | **Sì, mi sono già dovuto confrontare con questa situazione e …** | | | | | | | |
| --- | --- | --- | --- | --- | --- | --- | --- | --- | --- | --- | --- |
|  |  |  |  | **non mi ha affatto stressato/a** | | **mi ha un po’ stressato/a** | | **mi ha stressato/a abbastanza** | | **mi ha molto stressato/a** | |
| 1. Paziente in fin di vita isolato socialmente o dal punto di vista familiare | | 0 | | 1 | | 2 | | 3 | | 4 | |
| 1. Collega che non svolge correttamente il suo lavoro | | 0 | | 1 | | 2 | | 3 | | 4 | |
| 1. Mancanza di riconoscenza (da parte del paziente, della famiglia, dell’equipe, della gerarchia) | | 0 | | 1 | | 2 | | 3 | | 4 | |
| 1. Informazioni contraddittorie date alla famiglia da altre figure professionali sanitarie | | 0 | | 1 | | 2 | | 3 | | 4 | |
| 1. Presa in carico di pazienti giovani o con figli piccoli | | 0 | | 1 | | 2 | | 3 | | 4 | |
| 1. Mancanza di letti in reparto | | 0 | | 1 | | 2 | | 3 | | 4 | |
| 1. Difficoltà psicologiche o emozionali dei familiari | | 0 | | 1 | | 2 | | 3 | | 4 | |
| 1. Spazi di cura inadatti, carenza di organico o materiale difettoso | | 0 | | 1 | | 2 | | 3 | | 4 | |
| 1. Disaccordo o mancanza di coordinamento con gli altri reparti per la presa in carico del paziente | | 0 | | 1 | | 2 | | 3 | | 4 | |
| 1. Conflitto o disaccordo nell’ambito della famiglia riguardo la presa in carico del paziente | | 0 | | 1 | | 2 | | 3 | | 4 | |
| 1. Afflusso troppo importante di professionisti attorno al malato in una situazione di urgenza | | 0 | | 1 | | 2 | | 3 | | 4 | |
| 1. Somministrazione di cure incomprensibili o inutili in relazione alla situazione del paziente | | 0 | | 1 | | 2 | | 3 | | 4 | |
| 1. Difficoltà a trovare il mio ruolo, a far riconoscere le mie competenze e/o il mio parere nell’ambito dell’equipe | | 0 | | 1 | | 2 | | 3 | | 4 | |
| 1. Ambiente rumoroso | | 0 | | 1 | | 2 | | 3 | | 4 | |
| 1. Assenza di sostegno da parte dell’amministrazione | | 0 | | 1 | | 2 | | 3 | | 4 | |
| 1. Rischio di errore, paura di fare male il mio lavoro | | 0 | | 1 | | 2 | | 3 | | 4 | |
| 1. Cattiva atmosfera nell’ambito dell’equipe (pettegolezzi, voci, ecc.) | | 0 | | 1 | | 2 | | 3 | | 4 | |
| 1. Dover somministrare rapidamente delle cure in situazione di urgenza | | 0 | | 1 | | 2 | | 3 | | 4 | |
| 1. Paziente che si lamenta, che pone molteplici domande | | 0 | | 1 | | 2 | | 3 | | 4 | |
| 1. Ritmo o orari di lavoro difficilmente compatibili con la vita di famiglia o la vita sociale | | 0 | | 1 | | 2 | | 3 | | 4 | |
| 1. Conflitti con i membri dell’equipe | | 0 | | 1 | | 2 | | 3 | | 4 | |
| 1. Non poter comunicare con il paziente | | 0 | | 1 | | 2 | | 3 | | 4 | |
| 1. Cambiamento dei turni, prestazioni straordinarie | | 0 | | 1 | | 2 | | 3 | | 4 | |
| 1. Lavorare mentre si stanno vivendo momenti personali difficili | | 0 | | 1 | | 2 | | 3 | | 4 | |
| 1. Paziente che peggiora in maniera inattesa o inspiegabile | | 0 | | 1 | | 2 | | 3 | | 4 | |
| 1. Famiglia le cui convinzioni o il cui modo di vita sono in contraddizione con i miei valori o con il funzionamento del reparto | | 0 | | 1 | | 2 | | 3 | | 4 | |
| 1. Cattiva comprensione da parte della famiglia della gravità della diagnosi o della prognosi del paziente | | 0 | | 1 | | 2 | | 3 | | 4 | |
| 1. Serie di decessi di pazienti nel reparto in un arco limitato di tempo | | 0 | | 1 | | 2 | | 3 | | 4 | |
| 1. Paziente che mi fa pensare a uno dei miei parenti o a me stesso | | 0 | | 1 | | 2 | | 3 | | 4 | |
| 1. Impotenza o incompetenza nell’accompagnamento delle famiglie | | 0 | | 1 | | 2 | | 3 | | 4 | |
| 1. Cambiamento nella somministrazione delle cure o del progetto terapeutico in funzione del medico responsabile del paziente | | 0 | | 1 | | 2 | | 3 | | 4 | |
| 1. Mancanza di personale | | 0 | | 1 | | 2 | | 3 | | 4 | |
| 1. Paziente non collaborativo, aggressivo o delirante | | 0 | | 1 | | 2 | | 3 | | 4 | |
| 1. Morte di un paziente con il quale avevo instaurato un legame particolare | | 0 | | 1 | | 2 | | 3 | | 4 | |
| 1. Famiglia che non ha fiducia in me o nell’equipe | | 0 | | 1 | | 2 | | 3 | | 4 | |
| 1. Sovraccarico di lavoro importante e permanente | | 0 | | 1 | | 2 | | 3 | | 4 | |
| 1. Dover effettuare dei compiti che non conosco o per i quali manco di competenze | | 0 | | 1 | | 2 | | 3 | | 4 | |
| 1. Essere valutato o giudicato da altri membri dell’equipe | | 0 | | 1 | | 2 | | 3 | | 4 | |
| 1. Tempo dedicato a incombenze di tipo amministrativo sottratto a quello dedicato alle cure | | 0 | | 1 | | 2 | | 3 | | 4 | |
| 1. Presa in carico di patologie gravi o complesse | | 0 | | 1 | | 2 | | 3 | | 4 | |
| 1. Occuparmi di un paziente le cui cure non sono di competenza del reparto di Rianimazione | | 0 | | 1 | | 2 | | 3 | | 4 | |
| 1. Incertezza della diagnosi o del progetto terapeutico del paziente | | 0 | | 1 | | 2 | | 3 | | 4 | |
| 1. Mancanza di rispetto per il paziente (riguardo alle sue volontà, alla sua integrità, alla sua situazione, ecc.) | | 0 | | 1 | | 2 | | 3 | | 4 | |
| 1. Paziente che soffre fisicamente o psicologicamente | | 0 | | 1 | | 2 | | 3 | | 4 | |
| 1. Dover annunciare al paziente o alla famiglia una diagnosi di malattia grave o essere presente al momento dell’annuncio di una diagnosi di malattia grave | | 0 | | 1 | | 2 | | 3 | | 4 | |
| 1. Mancanza di equità nella distribuzione del lavoro tra colleghi | | 0 | | 1 | | 2 | | 3 | | 4 | |
| 1. Spazio di accoglienza per le famiglie inadatto o insufficiente | | 0 | | 1 | | 2 | | 3 | | 4 | |
| 1. Accumulo del carico di lavoro legato all’attività clinica, alla formazione, alla ricerca o all’insegnamento | | 0 | | 1 | | 2 | | 3 | | 4 | |
| 1. Prendere la decisione di interrompere o ridurre il trattamento | | 0 | | 1 | | 2 | | 3 | | 4 | |
| 1. Guardie o lavoro di notte | | 0 | | 1 | | 2 | | 3 | | 4 | |

1. Laurent A, Lheureux F, Genet M, Martin Delgado MC, Bocci MG, Prestifilippo A, et al. Scales Used to Measure Job Stressors in Intensive Care Units: Are They Relevant and Reliable? A Systematic Review. Front Psychol. 2020;11:245.

2. Field A. Discovering Statistics Using IBM SPSS statistics. 4th ed. London: Sage; 2013.

3. Reise SP, Yu J. Parameter Recovery in the Graded Response Model Using MULTILOG. J Educ Meas. 1990;27:133–44.

4. Hair JF, editor. Multivariate data analysis: a global perspective. 7. ed., global ed. Upper Saddle River, NJ: Pearson; 2010.

5. Guadagnoli E, Velicer WF. Relation of sample size to the stability of component patterns. Psychol Bull. 1988;103:265–75.

6. Comrey AL, Lee HB. A first course in factor analysis. 2016.

7. Bonett DG, Wright TA. Sample size requirements for estimating pearson, kendall and spearman correlations. Psychometrika. 2000;65:23–8.

8. Bonett DG. Sample Size Requirements for Testing and Estimating Coefficient Alpha. J Educ Behav Stat. 2002;27:335–40.

9. Bonett DG. Sample size requirements for estimating intraclass correlations with desired precision. Stat Med. 2002;21:1331–5.

1. Noisy environment [↑](#footnote-ref-1)
2. Not being able to communicate with the patient [↑](#footnote-ref-2)
3. Lack of respect towards the patient (towards his wishes, his integrity, his situation, etc.). [↑](#footnote-ref-3)
4. Unsuitable or under-equipped family reception area [↑](#footnote-ref-4)
